# Characterization of H type 1 and type 1 *N*-acetyllactosamine glycan epitopes on ovarian cancer specifically recognized by the anti-glycan monoclonal antibody mAb-A4

**DOI:** 10.1074/jbc.M116.768887

**Published:** 2017-02-06

**Authors:** Matthew Choo, Heng Liang Tan, Vanessa Ding, Roberto Castangia, Omar Belgacem, Brian Liau, Lauren Hartley-Tassell, Stuart M. Haslam, Anne Dell, Andre Choo

**Affiliations:** From the ‡ Department of Life Sciences, Imperial College London, London SW7 2AZ, United Kingdom,; the § Bioprocessing Technology Institute, Singapore 138668, Singapore,; the ‖ Institute for Glycomics, Griffith University, Southport, Queensland 4215, Australia, and; ¶ Shimadzu, Manchester M17 1GP, United Kingdom

**Keywords:** cancer biology, cancer therapy, embryonic stem cell, glycobiology, glycoconjugate, glycomics, mass spectrometry (MS), monoclonal antibody, ovarian cancer, small interfering RNA (siRNA)

## Abstract

Cancer-specific glycans of ovarian cancer are promising epitopes for targeting with monoclonal antibodies (mAb). Despite their potential, structural characterization of these glycan epitopes remains a significant challenge in mAb preclinical development. Our group generated the monoclonal antibody mAb-A4 against human embryonic stem cells (hESC), which also bound specifically to *N*-glycans present on 11 of 19 ovarian cancer (OC) and 8 of 14 breast cancer cell lines tested. Normal cell lines and tissue were unstained by mAb-A4. To characterize the *N*-linked glycan epitopes on OC cell lines targeted by mAb-A4, we used glycosidases, glycan microarray, siRNA, and advanced high sensitivity matrix-assisted laser desorption/ionization mass spectrometry (MALDI-MS). The mAb-A4 epitopes were found to be Fucα1–2Galβ1–3GlcNAcβ (H type 1) and Galβ1–3GlcNAcβ (type 1 LacNAc). These structures were found to be present on multiple proteins from hESC and OC. Importantly, endo-β-galactosidase coupled with MALDI-MS allowed these two epitopes, for the first time, to be directly identified on the polylactosamines of *N*-glycans of SKOV3, IGROV1, OV90, and OVCA433. Furthermore, siRNA knockdown of B3GALT5 expression in SKOV3 demonstrated that mAb-A4 binding was dependent on B3GALT5, providing orthogonal evidence of the epitopes' structures. The recognition of oncofetal H type 1 and type 1 LacNAc on OC by mAb-A4 is a novel and promising way to target OC and supports the theory that cancer can acquire stem-like phenotypes. We propose that the orthogonal framework used in this work could be the basis for advancing anti-glycan mAb characterization.

## Introduction

The discovery of novel surface antigens on ovarian cancer (OC)^4^ as therapeutic targets are in increasing demand because OC becomes resistant to chemotherapy in 80% of patients (1–6). A promising category of antigens are the glycans on glycoproteins or glycolipids, which have functional roles in cancer progression (7–19). These glycan antigens can be targeted by monoclonal antibodies (mAb), and indeed, several preclinical anti-glycan mAbs have been developed against OC and other cancers (20–27). However, very few glycan-specific anti-cancer mAbs have had their targets characterized in detail because of difficulties arising from the branched and non-template-driven nature of glycans (28). Not knowing the cancer epitope limits the clinical development of promising mAbs.

Recent efforts to characterize the *N*-glycan epitopes targeted by mAbs have used systematic knock-in and knockdown of glycosyltransferases to deduce their probable structures (20, 23) and also advanced mass spectrometry to detect potential *O-*glycan epitopes (21, 23). Although highly promising, these approaches were limited in their ability to provide unequivocal confirmation of target identities using tandem mass spectrometry due to the lack of diagnostic fragments (21, 23). This was due to insufficient cellular material because specific glycan epitopes are often a rare subset of the whole glycome (21, 23). Therefore, a strategy for enhancing the signal of low abundance cellular mAb targets is needed.

mAb-A4 was part of a panel of mAbs initially developed by immunizing mice with live pluripotent human embryonic stem cells (hESC) (29, 30). Because fetal antigens may be expressed on cancers (oncofetal antigens), it was hypothesized that the mAb-A4 against hESC might recognize cancers.

In this study, we characterized the binding of mAb-A4 to a panel of cancer and normal cell lines. Importantly, we elucidated the cellular glycan epitope responsible for mAb-A4 binding in ovarian cancer cell lines SKOV3, IGROV1, OV90, and OVCA433 using a multipronged orthogonal approach involving glycosidases, glycan microarray, siRNA knockdown, and advanced high sensitivity mass spectrometry of polyLacNAc. We report that the mAb-A4 epitopes on ovarian cancer cell lines are Fucα1–2Galβ1–3GlcNAcβ (H type 1) and Galβ1-3GlcNAcβ (type 1 LacNAc). These proposed epitopes were confirmed by diagnostic fragments in tandem mass spectrometry and also by siRNA knockdown of the galactosyltransferase gene *B3GALT5*.

## Results

### mAb-A4 binding to ovarian and breast cancer cell lines

FACS analysis found that mAb-A4 bound to hESC, 11 of 19 ovarian cancer cell lines, and 8 of 14 breast cancer cell lines studied (Table 1). In contrast, mAb-A4 did not bind to the normal human cell lines from foreskin fibroblast, lung fibroblast (IMR90), breast epithelial cells (MCF10A), immortalized ovarian surface epithelium (IOSE523), and mesenchymal stem cells from fetal, umbilical, and adult sources. This suggested that although the mAb-A4 antigen was not present on all ovarian or breast cancer cell lines, its expression was restricted to the cancer state.

**Table 1:** **mAb-A4 binding to cancer and normal cell lines on flow cytometry** Cells were stained with mAb-A4 followed by anti-mouse secondary antibody conjugated to FITC. Binding level was measured as relative increase in fluorescence compared with negative control as follows: −, negative; +, low; ++, medium; +++, high. Data are representative of three biological replicates.

Cell line	Binding
**hESC**	
HES-3	+++

**Ovarian cancer**	
OVCA433	+++
CH1	+++
HEY	+++
CaOV3	++
IGROV1	++
OV90	++
OVCAR3	++
PEA 1	++
SKOV3	++
HEY A8	+
HEY C2	+
A2780	−
COLO720E	−
OV17R	−
OVCAR8	−
OVCAR10	−
OVCA432	−
PEO 14	−
TOV112D	−

**Breast cancer**	
BT474	+++
MCF7	+++
CAMA1	+++
HCC2218	+++
HCC1954	+++
MB231	+++
MDA453	++
HCC1395	+
HCC1937	−
BT20	−
BT549	−
HS578T	−
T47D	−
SKBR3	−

**Normal**	
IOSE523 (ovary)	−
MCF10A (breast)	−
HFF (human foreskin fibroblast)	−
IMR90 (fibroblast)	−
Fetal MSC	−
Umbilical MSC	−
Adult MSC	−

**Figure 1.**
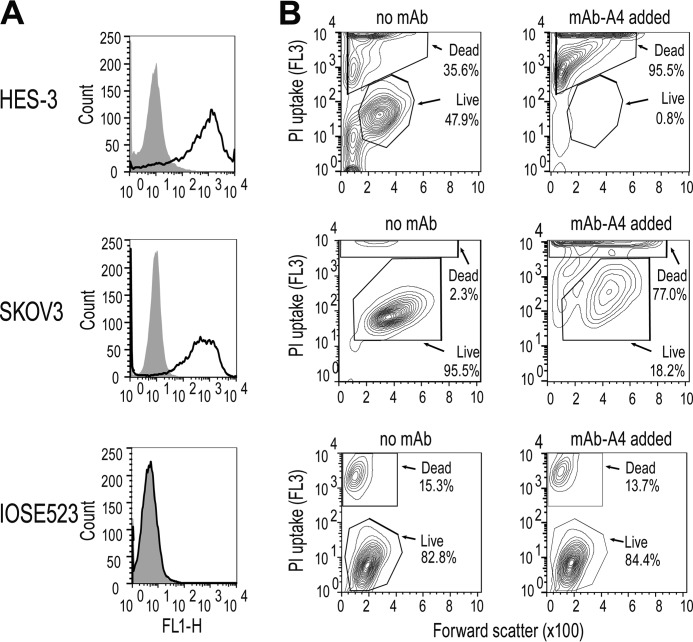
**Binding and cytotoxicity of mAb-A4 to cancer and normal and human embryonic stem cell lines.** *A,* mAb-A4 binding to HES-3 (*top*), SKOV3 (*middle*), and IOSE523 (*bottom*). Histograms of negative control (*filled*) were overlaid with mAb-A4-treated samples (*black line*). *B,* mAb-A4 cytotoxicity against HES-3 (*top*), SKOV3 (*middle*), and IOSE523 (*bottom*), showing untreated controls (*left column*) and mAb-treated samples (*right column*). Contour plots of forward scatter *versus* FL3 PI uptake were gated for live and dead populations based on the untreated cells. The data shown are representative of three biological replicates.

To characterize the mAb-A4 epitope, the ovarian adenocarcinoma cell line SKOV3 was chosen as the model cell line based on strong binding of the mAb in FACS, high growth rate, and available prior work on its glycome (10, 31). The mean fluorescence of mAb-A4 staining on hESC and SKOV3 on FACS was 2 orders of magnitude higher than the negative control (Fig. 1*A*). In contrast, the binding profile on IOSE523 was identical to the negative control, indicating that the epitope was not expressed on the surface or was inaccessible on IOSE523. Interestingly, mAb-A4 also had a direct cytotoxic effect against hESC and SKOV3 within 1 h of incubation, as measured by propidium iodide exclusion assay. After mAb-A4 was added, cell viability decreased from 47.9 to 0.83% in hESC and from 90.1 to 27.0% in SKOV3 (Fig. 1*B*). This cytotoxic effect of mAb-A4 was also seen in the high binding OC cell lines (Table 1). In contrast, mAb-A4 was not cytotoxic to IOSE523, consistent with the lack of binding to this cell line (Fig. 1).

### Immunohistochemistry

In an initial screen using tissue microarrays, mAb-A4 positively stained tumor tissue from breast, ovary, testis, lung, pancreas, bone, and small intestine but was unreactive with normal breast stroma, testis, liver, ovary, and skin (supplemental Fig. 1). Staining of adenocarcinoma of breast, lung, and small intestine was ductal, whereas staining of adenocarcinomas of ovary and pancreas was homogeneous. This initial screen suggested that mAb-A4 could potentially target malignant cells in a clinical setting.

### Glycan dependence of mAb-A4 binding

**Figure 2.**
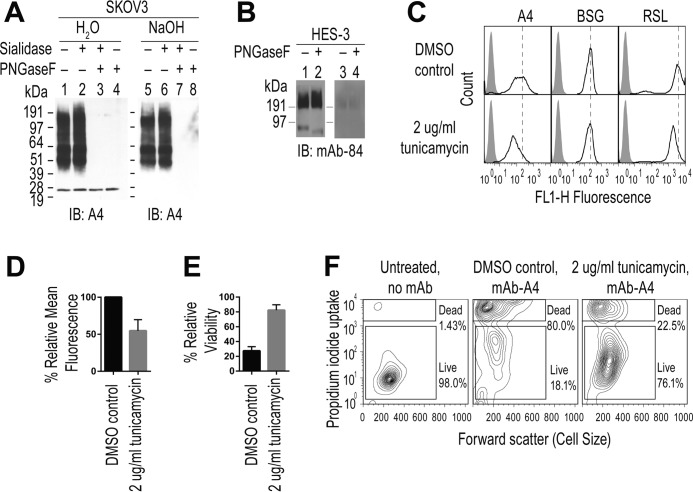
***N*-Glycan dependence of mAb-A4's binding to HES-3 and SKOV3.** *A,* SDS-PAGE Western blot of the mAb-A4 antigen immunoprecipitated from SKOV3 and digested with no enzyme (*lanes 1* and *5*), sialidase A (*lanes 2, 3, 6,* and *7*), PNGaseF (*lanes 3, 4, 7,* and *8*), or subjected to on-blot NaOH β-elimination (*lanes 5–8*), and immunoblotted (*IB*) with mAb-A4. *B,* SDS-PAGE Western blot of HES-3 lysate treated with no enzyme (*lanes 1* and *3*) or PNGaseF (*lanes 2* and *4*) and subjected to on-blot β-elimination (*lanes 3* and *4*), and immunoblotted with mAb-84. *C,* effect of 72 h of tunicamycin treatment on binding of mAb-A4, basigin (*BSG*), and biotinylated *R. solanacearum* lectin (*RSL*). Histograms show negative control (*filled*) overlaid with sample (*black line*). *Dashed vertical lines* show the mean fluorescence of the DMSO control without tunicamycin. *D,* effect of tunicamycin on the mean fluorescence of mAb-A4 binding to SKOV3. *E,* effect of tunicamycin on the cytotoxicity of mAb-A4 against SKOV3, as measured by relative viability through PI exclusion. *F,* effect of tunicamycin on the cytotoxicity of mAb-A4 against SKOV3. Contour plots of forward scatter *versus* PI uptake of FACS negative control (*left*), DMSO control (*middle*), and tunicamycin-treated (*right*). Data were representative of three biological replicates over successive passages. *Error bars* indicate one S.D.

**Figure 3.**
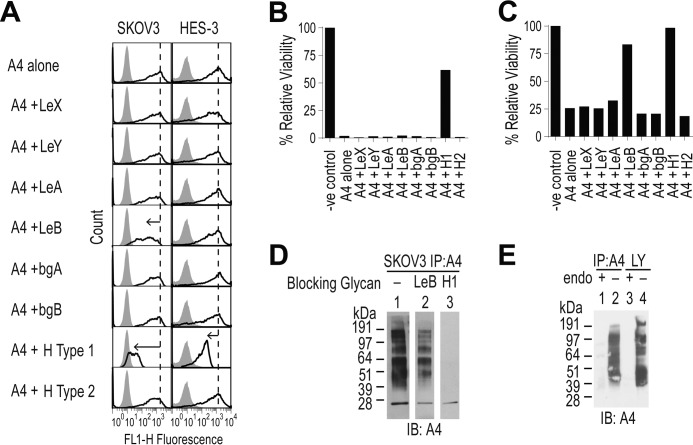
**Identifying potential epitopes targeted by mAb-A4.** *A,* inhibition of mAb-A4 binding to SKOV3 and HES-3 by a panel of soluble oligosaccharides. mAb-A4 was preincubated with 2 mm of the indicated oligosaccharide in PBS before being added to cells. Flow cytometry histograms showing negative control (*filled*) and mAb-A4 staining (*black line*). The *dashed vertical line* marks the mean fluorescence of the positive control, and the *black arrows* indicate shifts of mean fluorescence from the positive control. *B,* effect of 2 mm oligosaccharide preincubation with mAb-A4 on relative viability of HES-3, assayed by propidium iodide exclusion. *C,* effect of 2 mm oligosaccharide preincubation with mAb-A4 on relative viability of SKOV-3, assayed by propidium iodide exclusion. *D,* Western blot of immunoprecipitated mAb-A4 antigen from SKOV3 that was probed with mAb-A4 that had been preincubated with either PBS (*lane 1*), Lewis B (*lane 2*), or H type 1 (*lane 3*). *IB*, immunoblot. *E,* effect of endo-β-galactosidase (from *E. freundii*, “*endo*”) on mAb-A4 binding. SKOV3 whole cell lysate (*lanes 3* and *4*) and immunoprecipitated mAb-A4 antigen (*lanes 1* and *2*) were treated with endo-β-galactosidase (*lanes 1* and *3*) or an equivalent volume of water (*lanes 2* and *4*) and Western blotted with mAb-A4. Oligosaccharides used were Lewis X (*LeX*), Lewis Y (*LeY*), Lewis A (*LeA*), Lewis B (*LeB*), blood group A (*bgA*), blood group B (*bgB*), H type 1 (*H1*), and H type 2 (*H2*). All data were representative of three biological replicates.

The mAb-A4 antigen in SKOV3 was found by immunoprecipitation (IP) followed by Western blotting to be a smear from 40 to 191 kDa with more intense regions at 51, 60, and 100 kDa (Fig. 2*A, lanes 1* and *5*). This staining of IP product was similar to that of SKOV3 whole cell lysate (Fig. 3*E, lane 4*), suggesting that IP with mAb-A4 successfully enriched its antigen from the cell lysate. The presence of the smear suggested that the mAb-A4 antigen was a glycoprotein.

Removing *N*-glycans from the antigen using PNGaseF abolished mAb-A4 binding (Fig. 2*A, lanes 3, 4, 7,* and *8*). In contrast, mAb-A4 binding was unaffected by removal of sialic acid (Fig. 2*A, lanes 2* and *6*) or removal of *O-*glycans (*lane 5*). As a positive control for removal of *O-*glycans, mAb-84 binding to *O-*glycans of hESC was abolished by on-blot β-elimination, as expected (Fig. 2*B*) (30). In addition, tunicamycin treatment reduced mAb-A4 binding by almost half (mean fluorescence decreased by 45 ± 15%) and raised cell viability from 27.3 ± 5.7% to 82.0 ± 7.1% live cells (Fig. 2, *C–F*). Tunicamycin treatment also abolished the Western blotting signal for mAb-A4 (Fig. 11*F, lane 6*). Therefore, mAb-A4 binding was demonstrated to be dependent on *N*-glycans and not *O-*glycans.

### Oligosaccharide inhibition assay

To identify the unknown epitope, mAb-A4 binding to a panel of oligosaccharides was tested. Without any soluble oligosaccharide added, mAb-A4 bound to and was cytotoxic to HES-3 (<5% viable cells after 1 h) and SKOV3 (<30% viable cells after 1 h) (Fig. 3, *A–C*). This binding and cytotoxicity to HES-3 was blocked only by preincubation of mAb-A4 with the H type 1 trisaccharide, causing a 10-fold decrease in mean fluorescence and a rescue from <5 to 62% relative viability (Fig. 3, *A* and *B*). This inhibition by the H type 1 trisaccharide on binding and cytotoxicity was also observed for SKOV3 (100-fold decrease in mean fluorescence, rescued from 30 to 98% relative viability). When other fucosylated oligosaccharides were screened, only Lewis B tetrasaccharide had a blocking effect (10-fold decrease in mean fluorescence and rescued from 30 to 83% relative viability) (Fig. 3, *A* and *C*). In contrast, Lewis A, Lewis X, and Lewis Y had no effect on binding and cytotoxicity of mAb-A4 to SKOV3. Lewis B also partially blocked mAb-A4 on Western blotting of SKOV3 IP product (Fig. 3*D, lane 2*), whereas H type 1 completely blocked mAb-A4 (*lane 3*). Therefore, blocking was stronger when Fucα1–2 was present on Gal. In addition, because the glycans H type 2 and Lewis Y (both having type 2 LacNAc) were unable to block mAb-A4, we hypothesized that the type 1 LacNAc structure was also important to mAb-A4 binding. Comparing the strongly blocking H type 1 to the partially blocking Lewis B, adding Fucα1–4 to GlcNAc inhibited the binding, possibly by steric hindrance. Taken together, the preceding data suggested that type 1 LacNAc was critical to mAb-A4 binding and that α2-fucosylation of Gal improved binding, whereas fucosylation of GlcNAc hindered binding.

### Glycan microarray

**Figure 4.**
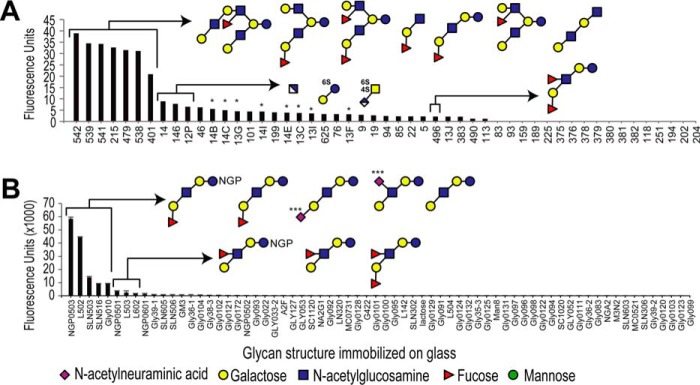
**Binding profile of mAb-A4 to two different glycan microarrays.** The hits were sorted by signal strength, and the two *arrowed brackets* indicate the strong and weak binders. *A,* mAb-A4 binding profile on a glycosylamine microarray detected by fluorescence of a secondary antibody. Only three of the low hits were *bracketed* and shown as schematics as the other low hits were variations of these three structures. *B,* mAb-A4 binding profile on a PNPA microarray detected by fluorescence of a secondary antibody. The top five hits and three of the low hits are *bracketed* and shown. Glycan numbers on the *horizontal axis* correspond to the list found in the supplemental material. *NGP*, neoglycoprotein presenting multiple glycan epitopes. *** indicates that partial desialylation had occurred, observed by HPLC. *White* and *blue square,* glucuronic acid; *4S* and *6S,* sulfation on C-4 and C-6.

To identify possible glycan epitopes, mAb-A4 was used to probe two different glycan microarrays. On the glycosylamine array, glycans that gave the highest signal had the common epitopes of type 1 LacNAc or H type 1 (Fig. 4*A*). The low binders were likely to be binding non-specifically. Lewis B, which blocked mAb-A4 binding at 2 mm, was a non-binder on this array. Similar results were obtained on a PNPA glycan microarray (Fig. 4*B*). The high binders were H type 1, followed by type 1 LacNAc. The signal produced by sialyl type 1 was due to the presence of contaminating (desialylated) type 1 LacNAc as determined by HPLC (data not shown). The very low binders were Lewis A and Lewis B and could be due to nonspecific binding. The results from two glycan microarrays with different attachment chemistries corroborated. We hypothesized that mAb-A4 was targeting H type 1 and, to a lesser extent, also type 1 LacNAc on *N*-glycans on ovarian cancer cells.

### MALDI-TOF N-glycome of SKOV3

**Figure 5.**
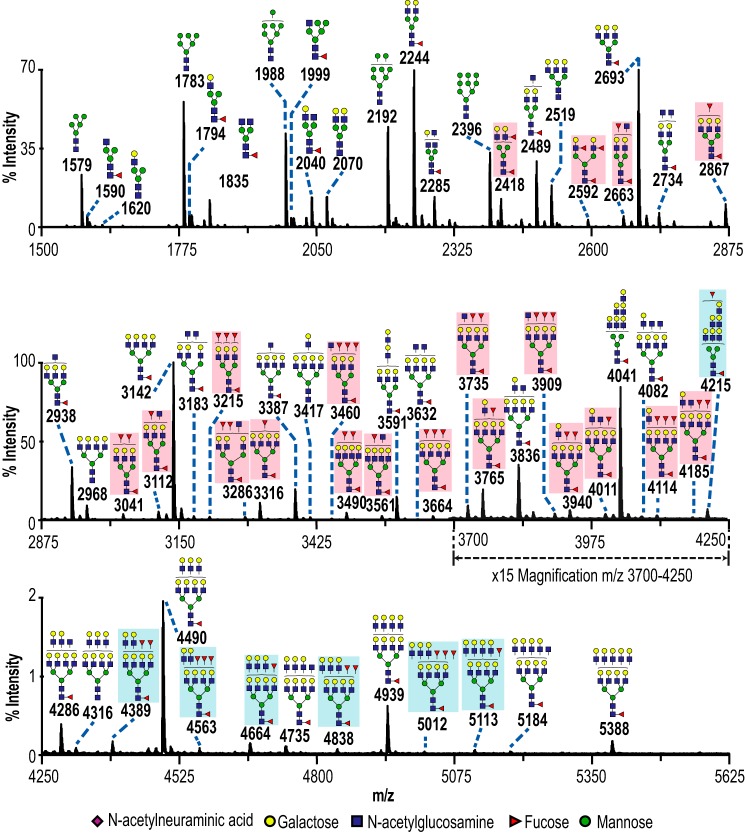
**Types of potential mAb-A4 epitopes in the *N*-glycome of SKOV3.** Partial mass spectrum of permethylated desialylated *N*-glycans from 10 million SKOV3 cells, acquired by MALDI-TOF in positive mode (50% acetonitrile fraction). Intensity was normalized to the *m/z* 3142 peak. The region from *m/z* 3700–4250 was magnified 15 times to show the minor peaks. *Red boxes* indicate outer-arm fucosylated *N*-glycans that lacked polyLacNAc antennae. *Blue boxes* indicate outer-arm fucosylated *N*-glycans with polyLacNAc antennae. Glycan residues shown outside of the *brackets* indicate ambiguity regarding their placement. Annotations of the polyLacNAc terminus were simplified to show the most probable permutation.

Next, the presence of H type 1 or type 1 LacNAc on ovarian cancer cell lines was investigated. To determine the cellular glycan target, total *N*-glycans were analyzed on MALDI-TOF as permethylated sodiated adducts in the positive mode (supplemental Fig. 3). Sialylated structures were more abundant than fucosylated structures and hindered identification of fucosylated structures by MS/MS. Therefore, to better identify potentially fucosylated structures, the *N*-glycans were desialylated (Fig. 5). The desialylated *N*-glycome consisted of high mannose and core-fucosylated complex-type *N*-glycans. Bi-, tri-, and tetra-antennary structures were observed. Outer-arm fucosylated glycans were present but were less abundant than non-fucosylated antennae. For example, the biantennary glycan at *m/z* 2244 was five times more intense than the *m/z* 2418 species with one antennal fucose (Fig. 5). This pattern was repeated for the tri- and tetra-antennary structures at *m/z* 2693/2867 and 3142/3316, respectively. This indicated that although there are active outer-arm fucosyltransferases in SKOV3, the fucosylation of antennae does not go to completion. No sialyl Lewis antigens were observed by MS/MS in the non-desialylated *N*-glycome (supplemental Fig. 3). In other words, antennae were either sialylated or fucosylated, but not both.

Glycans with potential polyLacNAc antennae were detected above *m/z* 3400 and were confirmed by MS/MS to have up to (LacNAc)_3_ (the *m/z* 1384 fragment ion). Sensitivity of these peaks to endo-β-galactosidase provided additional confirmation of polyLacNAc structures (supplemental Fig. 2).

### N-glycan sequencing by MALDI-TOF-TOF

**Figure 6.**
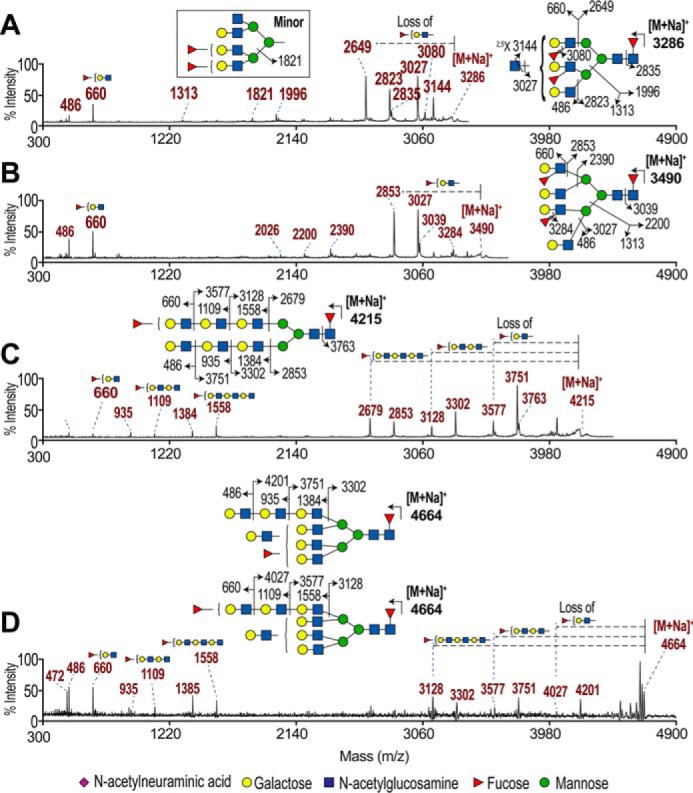
**Fragments from four precursors potentially carrying H type 1 from SKOV3 desialylated permethylated *N*-glycans.** *A,* MS/MS spectrum of *m/z* 3286 precursor. *B,* MS/MS spectrum of *m/z* 3490 precursor. *C,* MS/MS spectrum of *m/z* 4215 precursor. *D,* MS/MS spectrum of *m/z* 4664 precursor. Spectra were acquired by MALDI-TOF-TOF MS/MS using collision-induced dissociation at 1 kV. Small schematic annotation of peaks from the *left* indicated fucosylated LacNAc ions and from the *right* (*dotted arrows*) indicated their loss from the precursor ion. Glycan schematics indicate the major precursor ion, with *solid arrows* and *numbers* indicating observed fragmentation points and the resulting *m/z* of fragments. Note that which branch the antennae were on could not be assigned, but the schematics indicate a likely arrangement to annotate the points of fragmentation.

From the comprehensive MS/MS analysis done on many of the peaks, four representative glycans (*m/z* 3286, 3490, 4215, and 4664) were chosen to demonstrate the pool of potential H type 1 candidates. This pool was classified as short (Fig. 6, *A* and *B*) and extended (Fig. 6, *C* and *D*) based on the presence of fucosylated polyLacNAc antennae.

The non-reducing end *m/z* 660 fragment (composition of deoxy-HexHexHexNAc) confirmed that these glycans had potential H type 1 (FucGalGlcNAc) (Fig. 6). In addition, the lack of *m/z* 646 fragment (*m/z* 14 less than *m/z* 660) corresponding to internal FucGalGlcNAc in all four spectra indicated that the fucosylation was at the terminal LacNAc rather than an internal LacNAc.

For the short *N*-glycan precursors at *m/z* 3286 and 3490, upon MS/MS it was clear that both were core-fucosylated because of the loss of reducing end FucGlcNAc producing the *m/z* 2835 and 3039 ions, respectively (Fig. 6, *A* and *B*). Core fucosylation was consistent with the rest of the *N*-glycome. Terminal GlcNAc on the *m/z* 3286 precursor was identified by the loss of terminal GlcNAc (*m/z* 3144 and 3027 fragment). In both precursors, the lack of any difucosylated LacNAc (no *m/z* 834) and the presence of strong monofucosylated LacNAc (*m/z* 660) confirmed that the major species was one of the FucGalGlcNAc compositional isomers: Lewis A, Lewis X, H type 1, or H type 2. The *m/z* 3080 fragment was the elimination of fucose and water (−174−14−18 Da), suggesting that Fucα1–3GlcNAc from Lewis X was present. The lack of elimination of galactose and water indicated that Lewis A was not present. H type 1 was unlikely to be present because of lack of the elimination of Fucα1–2Gal from the C-3 position of GlcNAc (no peak at *m/z* 433), but its presence could not be excluded because this type of high energy fragmentation is uncommon for precursor ions of such high *m/z*.

In examining the extended *N*-glycan precursors at *m/z* 4215 and 4664 (Fig. 6, *C* and *D*), the spectra differed to that of the short *N*-glycans by the presence of two unique ion series. First, the *m/z* 486, 935, and 1384 non-reducing end fragments indicated antenna containing one, two, and three LacNAc, respectively (a difference of *m/z* 449 between fragments). Second, the corresponding reducing end fragments confirmed these losses of LacNAc as annotated: for the *m/z* 4215 precursor, fragments were observed at *m/z* 3751, 3302, and 2853. Likewise, for the 4664 *m/z* precursor, the fragments were observed at *m/z* 4201, 3751, and 3302.

In Fig. 6, *C* and *D*, the *m/z* 660, 1109, and 1558 non-reducing end fragments corresponded to one, two, and three LacNAcs plus fucose, respectively. As with the LacNAc series, the reducing end fragments were also present, as annotated by *dotted arrows* (Fig. 6, *C* and *D*). In the *m/z* 4215 precursor, these were *m/z* 3577, 3128, and 2679. In the *m/z* 4664 precursor, these were *m/z* 4027, 3577, and 3128.

Although the schematics in Fig. 6, *C* and *D,* show the polyLacNAc on the β1,6-GlcNAc antenna, this MS/MS experiment could not determine which antenna had the polyLacNAc. It is likely that there was a mixture of isobaric species with several permutations, and therefore the simplified annotated schematic shows the most important feature, the extended polyLacNAc with the ultimate LacNAc either undecorated or monofucosylated. On this information, the *N*-glycome was updated with a *red box* if only Fuc(LacNAc)_1_ was present and with a *blue box* if Fuc(LacNAc)_1–3_ were present (Fig. 5).

Although no FucGal fragment at *m/z* 433 diagnostic for H type 1 was observed, the MS/MS spectra of *m/z* 4215 and 4664 indicated that H type 1 was potentially on polyLacNAc of the larger *N*-glycans (Fig. 6, *C* and *D*). The short *N*-glycans were likely to be mostly Lewis X (Fig. 6, *A* and *B*). To test whether H type 1 was on the short *N*-glycans, MS3 with a MALDI-quadrupole-ion-trap-time-of-flight (MALDI-QIT-TOF) was used to further fragment the *m/z* 660 (FucGalGlcNAc) daughter fragments from the *m/z* 2418 and 2592 precursors. Diagnostic ions and ratios of ions were used to match the sample to a library of known structures (H type 1, Lewis A, and Lewis X). Only Lewis X was detectable on the short *N*-glycan but the *m/z* 433 fragment for H type 1 was not detected (supplemental Fig. 5).

To test whether the mAb-A4 antigen was on the polyLacNAc antennae, IP product and cell lysate was digested with endo-β-galactosidase from *Escherichia freundii* (Fig. 3*E*). Compared with the strong staining of mAb-A4 of the negative control IP product and cell lysate (Fig. 3*E, lanes 1* and *3*), the mAb staining of endo-β-galactosidase-treated samples was completely abolished (*lanes 2* and *4*). This demonstrated that the mAb-A4 antigen was present exclusively on the polyLacNAc antennae in SKOV3.

### PolyLacNAc termini of SKOV3 and IOSE523

**Figure 7.**
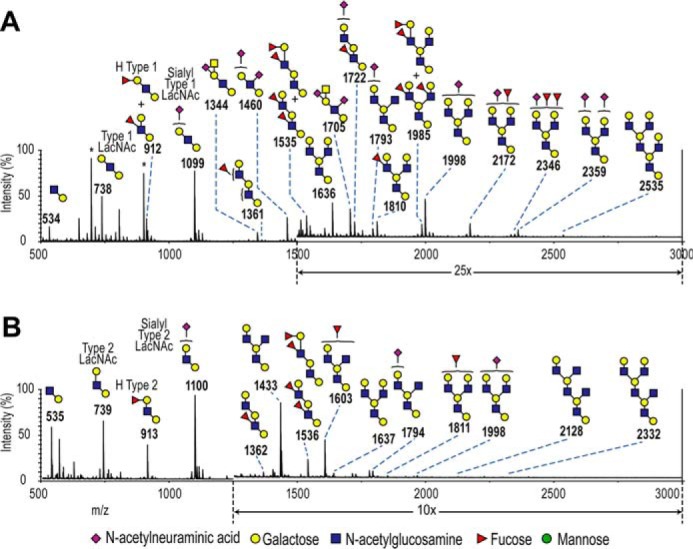
**Comparison of polyLacNAc termini released by endo-β-galactosidase from SKOV3 and IOSE523.** *A,* mass spectrum of the reduced permethylated termini from SKOV3 (35% acetonitrile fraction). The *m/z* 1500–3000 range was magnified ×25 to visualize the minor peaks. *B,* mass spectrum of the deuteroreduced permethylated termini from IOSE523 (35% acetonitrile fraction). The *m/z* 1250–3000 range was magnified ×10 to visualize the minor peaks. Spectra were acquired as sodiated adducts by MALDI-TOF in the positive mode. *Asterisks* indicate contaminant peaks.

To identify which FucGalGlcNAc isomer was present on polyLacNAc, SKOV3 and IOSE523 tryptic glycopeptides were digested with endo-β-galactosidase, and the released termini from SKOV3 and IOSE523 were analyzed by MALDI-TOF (Fig. 7). Here, IOSE523 was a mAb-A4 negative cell line for comparison.

As seen in Fig. 7, the released termini were found to be varied, with up to 19 glycan peaks identified, ranging from two residues (*m/z* 535) to 11 residues (*m/z* 2536). Importantly, potentially H type 1-fucosylated LacNAc glycans were present at *m/z* 913 (FucGalGlcNAcGal) and *m/z* 1362 (FucGalGlcNAcGalGlcNAcGal). In addition, type 1 LacNAc was potentially present at *m/z* 739 (GalGlcNAcGal). These precursors were of six or fewer residues and hence were prime candidates for comprehensive MS/MS linkage analysis. The glycans identified in the released termini were consistent with the known activity of endo-β-galactosidase, *i.e.* the reducing end Gal was a result of glycosidic cleavage of Gal from internal LacNAc, and resistance to the enzyme was conferred by polyLacNAc I-branching (GlcNAcβ1–6Gal) or fucosylation of the preceding GlcNAc (32, 33).

To assist in determination of linkages, schematics illustrating the diagnostic fragment ions for each isomer were made by manually by calculating all the possible fragmentation patterns (Fig. 9). The list of ions with their mathematical derivation can be found in supplemental Table 3. Then, the released termini with potential H type 1 and type 1 LacNAc were fragmented in MS/MS. MS/MS conducted on the *m/z* 739 precursors from SKOV3 and IOSE523 produced the *m/z* 486 non-reducing end HexHexNAc fragment, confirming the general GalGlcNAcGal structure (Fig. 9, *A* and *B*). Focusing on SKOV3, the strong *m/z* 503 reducing end HexNAcHex-itol fragment was the elimination of galactose and water, characteristic of substituents at the C-3 position of GlcNAc (34, 35). In contrast, the *m/z* 521 fragment was very minor in SKOV3, and this fragment is from the glycosidic cleavage of galactose without loss of water from the C-4 position of GlcNAc. The “elimination ratio” of the intensities of the eliminated fragment at *m/z* 503 to cleaved fragment at *m/z* 521 was 29.53. Furthermore, the elimination of galactose and water in conjunction with the ^0,4^X_GlcNAc_ cross-ring fragmentation resulting from a retro-Diels-Alder mechanism produced the *m/z* 429 fragment that was unique for type 1 (annotated as Z^0,4^X). The high elimination ratio and the strong Z^0,4^X fragment indicated that Galβ1–3GlcNAcβ1–4Gal (type 1 LacNAc) was the major species in the *m/z* 739 precursor from SKOV3 released termini.

**Figure 8.**
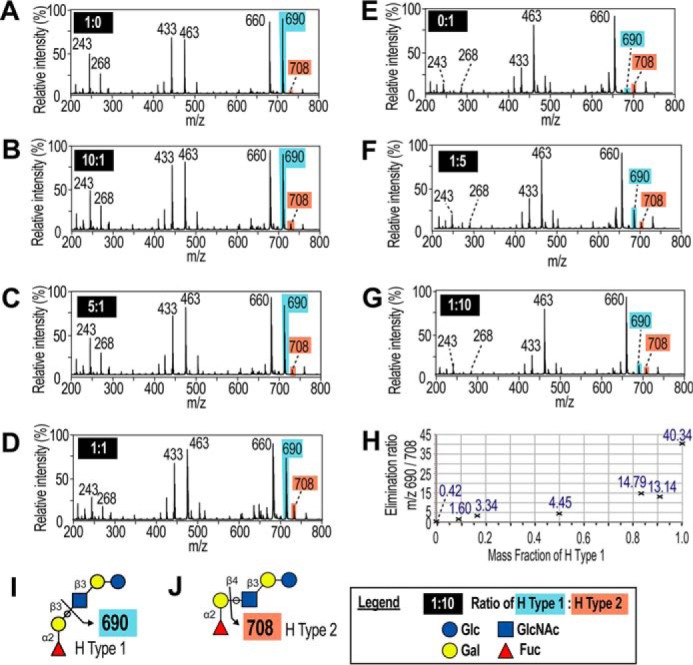
**Validation of the elimination ratio using known mixtures of LNFP1/H type 1 and LNnFP1/H type 2.** Glycan standards LNFP1 and LNnFP1 were mixed in the mass ratios indicated in the *black boxes. A–G,* mixtures were permethylated, and MALDI-TOF-TOF was used to acquire MS2 of the *m/z* 1100 precursor ion. *H,* elimination ratio was calculated from the intensity of the *m/z* 690 divided by *m/z* 708 ions and was plotted against the mass fraction of H type 1 *versus* H type 2. *I* and *J,* diagnostic fragmentation diagram from LNFP1/H type 1 and LNnFP/H type 2, respectively.

To confirm the use of the elimination ratio for discriminating between type 1 and type 2, the pentasaccharide glycan standards LNFP1 (H type 1) and LNnFP1 (H type 2) were mixed in different ratios and were fragmented in MS2 (Fig. 8). An elimination ratio above unity was indicative of the presence of type 1, whereas a ratio below unity was indicative of the absence of type 1. The SKOV3 elimination ratio of 29.53 corresponded with more than 90% H type 1.

**Figure 9.**
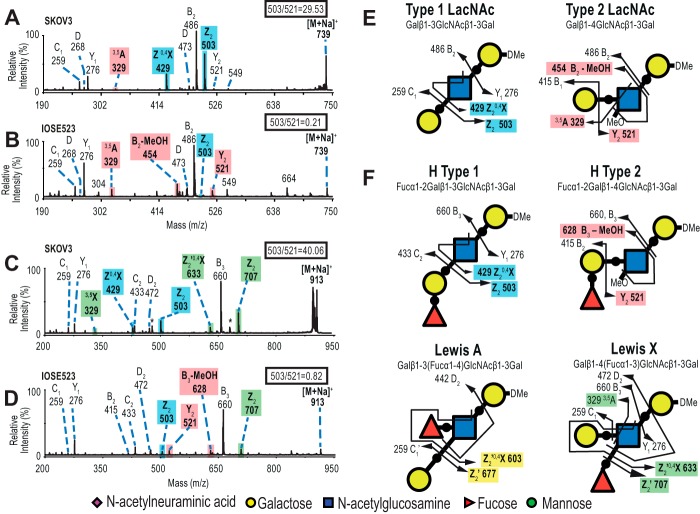
**Diagnostic fragments of blood group antigen isomers from polyLacNAc termini from SKOV3.** *A* and *B,* CID MS/MS of the *m/z* 739 precursor from SKOV3 and IOSE523. *C* and *D,* CID MS/MS of the *m/z* 913 precursor from SKOV3 and IOSE523. Permethylated and deuteroreduced glycans were analyzed by MALDITOF-TOF in the positive mode. The ratios of intensities of the *m/z* 503 and 521 peaks are indicated in *inset boxes. E,* fragmentation diagrams for the type 1 and type 2 LacNAc isomers. *F,* fragmentation diagrams for Lewis X, Lewis Y, H type 1, and H type 2 LacNAc isomers. Diagnostic ions are shaded according to the isomer of origin: *blue,* type 1 LacNAc; *red,* type 2 LacNAc; *yellow,* Lewis A; and *green,* Lewis X.

In contrast to SKOV3, the MS/MS of the *m/z* 739 precursor IOSE523 produced almost no *m/z* 503 fragment but a strong *m/z* 521 fragment (elimination ratio = 0.21) and no *m/z* 429 Z^0,4^X fragment (Fig. 9*B*). This indicated that the IOSE523 precursor at *m/z* 739 had no type 1 LacNAc. Instead, the strong *m/z* 454 fragment was produced by the loss of methanol (*m/z* 32) from C-3 of the GlcNAc from the GalGlcNAc fragment at *m/z* 486 (annotated as B_2_-MeOH). This suggested that the C-3 position of GlcNAc was not occupied by galactose but by a CH_3_O group (36). This group is indicative of the type 2 LacNAc (Galβ1–4GlcNAc). Additional evidence for type 2 LacNAc was that a strong *m/z* 329 was produced by a retro-Diels-Alder reaction to form the Gal^3,5^A_GlcNAc_ cross-ring fragment that is unique to Galβ1–4GlcNAc. This indicated that type 2 LacNAc was the major glycan species in the *m/z* 739 precursor from IOSE523.

This result was paralleled in the *m/z* 913 FucGalGlcNAcGal precursor from SKOV3 and IOSE523 (Fig. 9, *C* and *D*). The elimination ratio in SKOV3 was 40.06, whereas in IOSE523 it was 0.82, indicating predominant H type 1 in SKOV3 and lack of H type 1 in IOSE523 (Fig. 8). Also, the *m/z* 429 Z^0,4^X fragment was strong in SKOV3 and absent from IOSE523, whereas the *m/z* 628 B_3_-MeOH fragment was absent from SKOV3 but strong in IOSE523. These two pieces of evidence indicated that H type 1 was present in the *m/z* 913 precursor in SKOV3, whereas IOSE523 had predominantly H type 2 and undetectable levels of H type 1.

It should be noted that in the SKOV3 MS/MS of the *m/z* 913 precursor, some Lewis X was detectable by the following: 1) the presence of the Z_2_ *m/z* 707 fragment corresponding to elimination of fucose with water; 2) the related *m/z* 633 Z_2_^0,4^X fragment; and 3) the cross-ring *m/z* 329 Gal^3,5^X_GlcNac_ fragment indicating that there was some Galβ1–4GlcNAc present. Therefore, there was also some Lewis X present on the polyLacNAc of both SKOV3 and IOSE523.

### Type 1 glycans on more OC cell lines

There was an initial correlation between mAb-A4 binding and the presence of H type 1 and type 1 LacNAc. This supported the hypothesis that mAb-A4 was binding to H type 1 and type 1 LacNAc on OC cell lines. To test if this correlation held across more OC cell lines, analysis of endo-β-galactosidase released termini was carried out on five other mAb-A4 FACS-positive OC cell lines (IGROV1, OV90, OVCA433, HEYA8, and OVCAR3) as well as another mAb-A4 FACS-negative OC cell line OVCAR8. These cell lines were chosen to represent a cross-section across an epithelial-mesenchymal transition scale and molecular subtyping system (see supplemental Table 2) (37, 38). To check whether the mAb-A4 epitope was present on polyLacNAc termini across the FACS-positive cell lines, Western blotting analysis showed a partial or complete loss of mAb-A4 signal after endo-β-galactosidase digestion (supplemental Fig. 4). This indicated that some if not all the mAb-A4 antigen would be present in the released termini.

**Figure 10.**
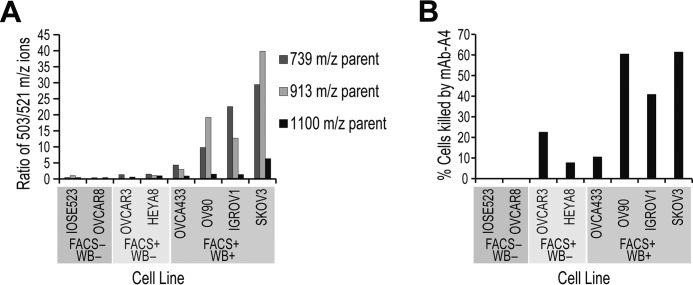
**Type 1 elimination ratio and mAb-A4 cytotoxicity across OC cell lines.** *A, column chart* showing the ratio of intensities of the *m/z* 503/521 fragment ions for three precursors: *m/z* 739 GalGlcNAcGal, *m/z* 913 FucGalGlcNAcGal, and *m/z* 1100 NeuAcGalGlcNAcGal. The cell lines were grouped into three groups based on staining with mAb-A4: FACS positive or negative, and Western blot positive or negative. Cell lines were arranged based on the elimination ratio of the *m/z* 739 precursor and their binding to mAb-A4 on FACS and Western blot. *B, column graph* showing the cytotoxicity of mAb-A4 against the OC cell lines, as measured by PI exclusion assay on FACS.

Positive mAb-A4 immunoblotting of cell lysate and positive FACS binding correlated with high elimination ratios of *m/z* 503/521 ions for the precursors at *m/z* 739, 913, and 1100 (Fig. 10*A*). In descending order of the *m/z* 739 precursor's elimination ratio were SKOV3 > IGROV1 > OV90 and OVCA433 > HEYA8 > OVCAR3. The two mAb-A4 negative cell lines IOSE523 and OVCAR8 had amounts of *m/z* 503 at the noise level, as reflected in their near-zero elimination ratios. Within each cell line, the abundances of H type 1 and type 1 LacNAc were comparable, which was reasonable because H type 1 is synthesized from type 1 LacNAc. Of the mAb-A4 FACS-positive cell lines, SKOV3, IGROV1, OV90, and OVCA433 showed strong mAb-A4 signal on Western blotting of whole cell lysate, but HEYA8 and OVCAR3 did not have any Western blotting signal (supplemental Fig. 4). This was consistent with the idea that if the total glycoproteins had low/undetectable type 1 LacNAc (observed in HEYA8 and OVCAR3), then they would not be expected to produce any signal on Western blotting (also observed in HEYA8 and OVCAR3).

Interestingly, in Fig. 10*B*, the cytotoxicity of mAb-A4 against the different cell lines appeared to correlate with the abundance of H type 1 (*m/z* 913 precursor), with the exception of OVCAR3. The moderate cytotoxicity of mAb-A4 against OVCAR3 but absent H type 1 signal was likely due to a non-proteinaceous antigen such as glycosphingolipid carrying type 1 LacNAc or H type 1, which also explained why the antigen was not visible on Western blot. Nevertheless, overall levels of type 1 LacNAc and H type 1 were strongly correlated with mAb-A4 cytotoxicity and Western blotting signal. This was consistent with the hypothesis that mAb-A4 targets H type 1 and type 1 LacNAc on OC cell lines.

### Expression of β3-galactosyltransferase in SKOV3

**Figure 11.**
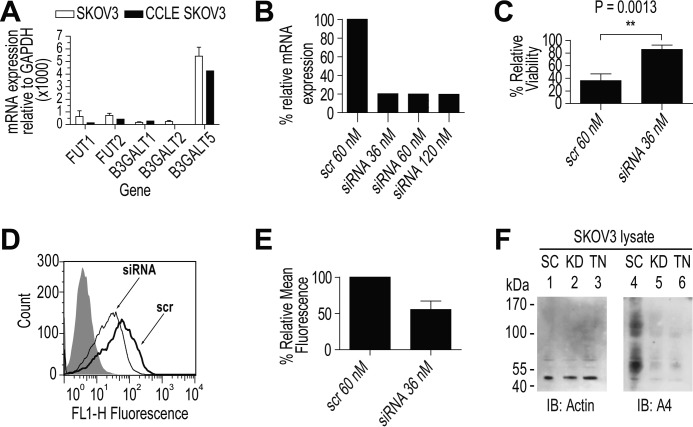
**Effects of siRNA knockdown of B3GALT5 in SKOV3.** Data were taken 72 h after siRNA was reverse-transfected into SKOV3. *A,* comparison of expression levels of genes responsible for H type 1 found in this study by qRT-PCR (*open columns*) *versus* data from the CCLE (*filled columns*) for SKOV3. *Error bars* show 1 S.D. from three biological replicates. *B,* effect of increasing concentration of siRNA *versus* scramble on mRNA of B3GALT5 measured by qRT-PCR and effect on cell yield as measured by hemocytometer. *C,* effect of siRNA knockdown on mAb-A4 cytotoxicity. Data were from three biological replicates (**, *p* = 0.0013). *D,* binding profile of mAb-A4 to SKOV3 transfected with 36 nm B3GALT5 siRNA or scramble (*scr*). *E,* change in relative mean fluorescence of mAb-A4 to SKOV3 transfected with 36 nm B3GALT5 siRNA or scramble (*scr*). Data were from three biological replicates. *F,* effect of 36 nm siRNA knockdown (*KD*) or 5 μg/ml tunicamycin (*TN*) on anti-β-actin and mAb-A4 Western blot of SKOV3 whole cell lysate, compared with scramble (*SC*). The samples were equally split for staining with anti-β-actin (42 kDa) and mAb-A4. Molecular mass markers in kDa are shown on the *left. IB*, immunoblot.

For an orthogonal confirmation of the observed type 1 LacNAc and H type 1, gene expression of the involved glycosyltransferases was measured in SKOV3. SKOV3 displayed strong expression of B3GALT5 and very weak expression of B3GALT1 and B3GALT2, consistent with the meta-analysis of the Cancer Cell Line Encyclopedia (CCLE) (Fig. 11*A*) (39). This suggested that B3GALT5 was the main galactosyltransferase responsible for the synthesis of type 1 LacNAc in SKOV3. The FUT1 and FUT2 expressions were both low, agreeing with the CCLE meta-analysis (Fig. 11*A*). These data were consistent with the observation in SKOV3 of type 1 LacNAc and Fucα1–2 of H type 1 (structures based on Galβ1–3GlcNAc).

### siRNA knockdown

Although type 1 LacNAc and H type 1 were observed on SKOV3 by MS/MS and supported by gene expression, direct evidence was needed that mAb-A4 was functionally binding these glycans. Compared with scramble siRNA, 36 nm siRNA against B3GALT5 was sufficient to reduce the mRNA of B3GALT5 by 80% after 72 h (Fig. 11*B*). This decrease in B3GALT5 mRNA was accompanied by the following: (i) a 73% reduction in mAb-A4 cytotoxicity (Fig. 11*C*); (ii) a 50% decrease in mean fluorescence of mAb-A4 binding (Fig. 11, *D* and *E*); and (iii) the reduction of mAb-A4 signal on a Western blot to the level of tunicamycin treatment (Fig. 11*F*). These data demonstrated that the mAb-A4 antigen was controlled by B3GALT5, and therefore, the antigen was dependent on Galβ1-3GlcNAc. Therefore, the knockdown data were consistent with mAb-A4 targeting H type 1 and type 1 LacNAc on OC cell lines.

### Shotgun proteomics

Proteins found in the IP product could be grouped into the following: 1) amino acid transport: 4F2/CD98hc, Basigin/CD147, neutral amino acid transporter B(0), sodium-coupled neutral amino acid transporter 2; 2) desmosome components: desmoglein-1, desmocollin-1, and Junction plakoglobin; 3) adhesins: podocalyxin and LAMP1; and 4) protein folding: heat shock protein 90b (supplemental Table 6).

## Discussion

mAb-A4 specifically recognizes ovarian cancer cells in a glycan-dependent manner. The terminal epitopes specifically recognized by mAb-A4 are proposed to be Galβ1–3GlcNAc (type 1 LacNAc) and Fucα1–2Galβ1–3GlcNAc (H type 1). Even though there were multiple bands stained by mAb-A4 on Western blotting of hESC and cancer cell lysate, these bands were different proteins with the common glycan epitope because the B3GALT5 knockdown abolished Western blot binding.

H type 1 and type 1 LacNAc were previously found to be pluripotency-associated antigens on hESC (40–42). Furthermore, the glycosyltransferase genes B3GALT5, FUT1, and FUT2 (which are responsible for type 1 LacNAc and H type 1) are up-regulated during induction from somatic to pluripotent stem cells (43–46). This suggested that B3GALT5 may be responsible for pluripotent markers based on type 1 LacNAc in hESC. Because type 1 LacNAc was found to be controlled by B3GALT5 in SKOV3, it is possible that B3GALT5 was being activated in OC in a similar way to pluripotent induced and embryonic stem cells. Furthermore, B3GALT5 is not expressed in the ovaries (47), suggesting that OC may up-regulate B3GALT5/type 1 LacNAc as part of disease progression. B3GALT5 has been shown to be controlled by DNA methylation, and this may be how hESC or OC activates B3GALT5 (48–50). Therefore, mAb-A4 appears to target a phenotype that is common between hESC and OC. This is consistent with the FACS screening data showing that mAb-A4 recognizes both hESC and OC cell lines.

It is intriguing that these two pluripotency markers are also associated with OC, supporting the theory that cancers can acquire stem-like phenotypes to their advantage (51–54). Indeed, teratomas generated from hESC with higher expression of H type 1 initially grew quicker than teratomas from hESC with lower expression of H type 1 (40). It was unknown whether H type 1 directly conferred the growth advantage or was just reflective of an internal state of higher pluripotency. In support of conferring a growth advantage, the B3GALT5 siRNA knockdown in this work appeared to suppress cell growth (Fig. 11*B*). However, measuring real time growth would be more definitive. Therefore, because H type 1 and type 1 LacNAc are pluripotent markers that may have a role in proliferation, mAb-A4 may have potential in therapy or diagnosis, subject to further screening for sensitivity and specificity against a larger set of clinical samples. It also remains to be explored whether H type 1 has biological significance when presented on the protein antigens identified by shotgun proteomics (supplemental Table 6).

Compared with previous mAbs targeting similar glycans, mAb-A4 appeared to differ in immunohistochemistry staining from RAV12 (26). Also, anti-HESCA-2 stains primarily a single 250-kDa band on Western blotting of hESC lysate (27), whereas mAb-A4 stains multiple bands on hESC, indicating that these mAbs target different epitopes (data not shown). FG-88 also did not react with OVCAR3 (22), whereas mAb-A4 did (Table 1). Anti-SSEA-5, reported as binding H type 1 trisaccharide (40), also failed to recognize SKOV3 and HEYA8 via FACS, whereas mAb-A4 bound strongly to them (supplemental Fig. 6). Therefore, mAb-A4 appeared to recognize a novel epitope that included type 1 LacNAc or H type 1 in OC.

To the best of our knowledge, this is the first report of H type 1 and type 1 LacNAc observed on polyLacNAc from SKOV3. The low levels of fucosylated blood groups detected here extend previous mass spectrometric studies on the *N*-glycome of SKOV3, OVCAR3, and IGROV1 that did not investigate fucosylated blood groups (9, 10, 31). Interestingly, in this work, mAb-A4's epitope was not identifiable even after extensive MS/MS of the total cell glycome. Instead, the type 1 glycan epitopes were only discovered after we scrutinized the polyLacNAc from SKOV3, guided by mAb-A4. This approach demonstrates that a mAb-centric search for cancer-associated glycosylation can indeed identify low abundance glycan epitopes on *N*-glycans, epitopes that are otherwise undetectable by screening or by whole cell glycomics. As mass spectrometric sensitivity continues to be improved, such low abundance epitopes may emerge as important in detecting and targeting malignancy, as in the case of mAb-A4. The ability of mAb-A4 to recognize cancer cells suggests that despite having low abundance, minor glycan peaks can be important for the discrimination between cancer and normal cells.

Characterizing the cellular target of anti-glycan mAbs in greater detail gives a clearer picture of the mAb's specificity and clinical potential. Based on this work, we propose a workflow to identify the cellular glycan epitope of an anti-glycan mAb (supplemental Fig. 7). First, the glycan type needs to be identified through chemical or enzymatic degradation on Western blotting and then confirmed using inhibitors of glycosylation. Second, potential epitopes should be identified by glycan microarray. Third, potential glycan targets should be identified in the cellular *N*- or *O-*glycome and confirmed with gene expression data. Informed by the glycome, the search space should be narrowed down using glycosidases. The linkages of the potential glycan epitope should be identified (such as analyzing polyLacNAc termini) to give direct proof of existence on the cell. Such direct proof is often lacking in mAb characterization studies. Finally, mAb binding to the hypothesized epitope should be functionally verified by knockdown of the appropriate glycosyltransferase in a live cell-based assay.

## Experimental procedures

### Cell culture

Ovarian cancer cell lines were chosen to give a cross-section across the EMT scale the and five molecular subtyping system (supplemental Table 2) (37, 38). SKOV3 was cultured in DMEM high glucose/DMEM low glucose (1:1 mix) (Gibco, Life Technologies, Inc.) with 10% fetal bovine serum (HyClone, GE Healthcare). The cell lines IGROV1, HEYA8, and OVCAR8 were cultured in RPMI 1640 medium (Gibco, Life Technologies, Inc.) with 10% FBS. OVCAR3 was cultured in RPMI 1640 medium with 20% FBS. IOSE523 and OV90 were cultured in MCDB105/M199 (1:1 mix) (Sigma) with 10% FBS. OVCA433 was cultured in DMEM high glucose (Gibco, Life Technologies, Inc.). The hESC line HES-3 (from ES Cell International Pte. Ltd., Singapore) was cultured as clumps on Matrigel (Corning Glass) in conditioned media from mouse embryonic fibroblasts (29). mAb-A4 hybridoma was cultured in Medium E (Stemcell Technologies). Ovarian cancer cell lines were obtained from the Cancer Science Institute of Singapore, National University of Singapore, Singapore, as reported previously (38).

### Antigen purification

Cells were trypsin-harvested and lysed in 1% (v/v) Triton X-100/PBS (Bio-Rad). The mAb-A4 antigen was immunoprecipitated from cell lysate by affinity chromatography using biotinylated mAb-A4 on an automated Phynexus MEA system (Phynexus, Inc.) and eluted in 100 mm sodium phosphate, pH 2.5 (29).

### Glycan standards

The following glycan standards were used: the trisaccharides Lewis A, Lewis X, blood group A, blood group B, H type 1, H type 2; the tetrasaccharides Lewis B and Lewis Y; and the pentasaccharides LNFP1 and LNFP 2 (Dextra Laboratories, Reading, UK); type 1 tetrasaccharide and LNnFP1 (Oligotech-Elicityl, France) (for structures, see supplemental Table 1).

### Antibodies and lectins

The murine IgM mAb-A4 hybridoma was derived from inoculating BALB/c mice with live HES-3, a human embryonic stem cell line (ES Cell International, Singapore), as described previously (29). mAb-A4 was selected based on its strong reactivity with HES-3 and cancer cell lines and non-binding to normal cell lines. mAb-A4 was purified from hybridoma supernatant using hydroxyapatite chromatography (55). The IgM mAb-84, targeting H type 1 on *O-*glycans of podocalyxin, was obtained as described previously (29, 30).

The purchased primary mAbs were as follows: murine IgG anti-basigin/CD147 (Santa Cruz Biotechnology); murine IgG anti-SSEA-5 (Millipore, MA); and murine IgG3 anti-H type 1 mAb 17-206 from ascites (Abcam, MA). Secondary probes were goat anti-mouse antibodies conjugated to horseradish peroxidase (HRP), goat anti-mouse antibodies conjugated to fluorescein isothiocyanate (FITC), streptavidin conjugated to HRP, and streptavidin conjugated to FITC (Dako, Glostrup, Denmark), and goat anti-mouse-IgM antibodies conjugated to Alexa Fluor 488 (Thermo Fisher Scientific). Lectins used were biotinylated *Ralstonia solanacearum* lectin (bRSL) (binds *N*-glycan core fucose) (56), a gift from the Imberty lab, CERMAV, France; and biotinylated *Datura stramonium* agglutinin (bDSA) (binds LacNAc) from Vector Laboratories.

### Tunicamycin treatment of SKOV3

SKOV3 was grown in complete media with 2.0 or 5.0 μg/ml tunicamycin (Sigma) dissolved in DMSO (Sigma) or an equivalent volume of DMSO for 72 h. SKOV3 cells were then trypsin-harvested and subjected to FACS analysis or were lysed and Western-blotted with mAb-A4.

### mAb binding and cytotoxicity assay

Cells were trypsin-harvested, washed in 1% BSA/PBS, incubated for 30 min on ice with neat mAb-A4 supernatant, 5 μg of anti-SSEA-5, 10 μl of mAb 17-206, 5 μl of anti-basigin or 5 μg of bRSL, or 1% BSA/PBS as negative control, and washed. Cells were then incubated for 15 min with a 1:500 dilution of goat anti-mouse antibody or streptavidin conjugated to FITC (Dako, Glostrup, Denmark) and washed again. Cytotoxicity was measured by propidium iodide exclusion (PI, 1.25 mg/ml). Data were acquired on FACSCalibur (BD Biosciences) and analyzed with FlowJo software (FlowJo, LLC). Live cells were gated as the PI low population in the negative control. Viability was calculated as the number of gated live cells divided by the total event count, and relative viability was the viability of the sample divided by the viability of the negative control.

### Oligosaccharide inhibition assay

To determine the terminal epitope recognized by mAb-A4, a panel of eight glycan standards were used to block mAb-A4 from binding cells on FACS by preincubation with primary antibody for 15 min (final oligosaccharide concentration of 2 mm). As a negative control, PBS with no glycan standard was used. A mAb-A4 and glycan standard mixture was used to probe Western blottings of cell, followed by HRP-conjugated anti-mouse secondary antibodies.

### Enzymatic digests

Cell lysate or IP product was denatured and reduced at 95 °C for 10 min with 0.2% SDS, 0.25% DTT. Samples were subjected to a combination of three treatments. 1) Sialic acids were removed with 5 milliunits of α2–3,6,8,9-neuraminidase (*Arthrobacter ureafaciens,* recombinant in *Escherichia coli*, Calbiochem) overnight at 37 °C in sodium phosphate, pH 6. As negative controls, an equal volume of water was added instead of enzyme. 2) *N*-Glycans were removed by incubating with 1000 units of PNGaseF (*Flavobacterium meningosepticum* recombinant in *E. coli*, New England Biolabs). As negative controls, an equal volume of water was added instead of enzyme. 3) *O-*Glycans were removed from proteins blotted on PVDF membrane by β-elimination by an overnight treatment in 50-ml centrifuge tubes with 50 mm NaOH (Sigma) at 42 °C, as described previously (57). As negative controls, blots were incubated with water in identical conditions. PVDF membranes were then washed thoroughly with PBS and then were blocked and probed as normal. To test whether the mAb-A4 antigen was presented on polyLacNAc, mAb-A4 IP product or cell lysate was digested with 40 units of sialidase A (*A. ureafaciens*, recombinant *E. coli*, New England Biolabs) and 25 milliunits of endo-β-galactosidase (*E. freundii*, Seikagaku Corp., Japan) in 100 mm sodium acetate, pH 5.8, for 16 h at 50 °C. As negative controls, sodium phosphate buffer was added instead of enzyme.

### Western blotting

Whole cell lysate or IP product was resolved in 4–12% (w/v) BisTris acrylamide gels (Life Technologies, Inc.) by denaturing SDS-PAGE and transferred to PVDF membrane (Bio-Rad), blocked in 5% BSA/PBS for 30 min, probed with primary antibody for 1 h at room temperature, washed three time for 5 min with PBS with 0.1% Tween 20 (PBST), followed by HRP-conjugated secondary antibodies for 1 h at room temperature, washed three times in PBST, and detected by the ECL chemiluminescent method using photographic film (GE Healthcare).

### Shotgun proteomics

IP product was resolved on SDS-PAGE, and gel bands corresponding to bands on Western blots were excised. Proteins were reduced, alkylated, and trypsinized, and peptides were acquired by C18 reversed phase chromatography coupled to electrospray ionization mass spectrometry with a Thermo Orbitrap Fusion. Proteins were identified using Proteome Discoverer with a minimum of two unique peptides, and were filtered to be smaller than the migration on a Western blot and to have putative *N*-glycosylation sites according to UNIPROT.

### Glycan microarray

The glycosylamine-epoxy-glass microarray slides were prepared and probed with 1 μg of mAb-A4, as described previously (58, 59). Positive binding of mAb-A4 to a particular glycan structure was when the relative fluorescence units of the replicate spots were three standard deviations above mean background and when the *p* value of spot replicates compared with background was below 0.005. A full list of glycan structures on the microarray is given in the supplemental material.

The PNPA glycan array consisted of various synthetic glycans with linkers immobilized on a glass surface and organized as triplicate spots. mAb-A4, diluted at either 5 or 2.5 μg/ml in PBS with 1 mm calcium and magnesium ions, was added to the glycan microarray and allowed to bind for 1 h. The spots were then washed with PBS and incubated with 1:10,000 dilutions of goat anti-mouse-IgM antibodies conjugated to Alexa Fluor 488 (Thermo Fisher Scientific) in PBS for 1 h and washed, and the fluorescence was measured using a Scanarray Gx microarray slide reader (PerkinElmer Life Sciences) in relative fluorescence units. Glycan spots were deemed positive binders if their signals were above the background noise for the triplicate spots at both 5 and 2.5 μg/ml mAb-A4. The positive binders were collated, and a common binding motif was deduced.

### Glycomic analysis by MALDI-TOF-TOF

The total cell *N*-glycome and *O-*glycome of cell lines were prepared as described previously (60, 61). *O-*Glycans were reduced with sodium borohydride or sodium borodeuteride (Sigma). Released glycans were permethylated with iodomethane and powdered sodium hydroxide (36, 62). Permethylated glycans were fractionated by Sep-Pak C18 Classic Cartridge into 15, 35, 50, and 75% acetonitrile fractions and analyzed by MALDI-TOF and MALDI-TOF-TOF on an ABSciex 5800, as described previously (36). Glycan compositional assignment was confirmed by extensive MS/MS fragmentation analysis with manual interpretation and reconstruction of the precursor ions using GlycoWorkBench (63).

### Isolation and analysis of polylactosamine terminal fragments by MALDI-TOF-TOF

Lyophilized tryptic glycopeptides from SKOV3, IGROV1, OV90, OVCA433, HEYA8, OVCAR3, OVCAR8, and IOSE523 were digested with endo-β-galactosidase (*E. freundii*, Seikagaku Corp., Japan) in 100 mm sodium acetate, pH 5.8, for 48 h, adding 1 5 milliunits at the start and another 15 milliunits at 24 h, and lyophilized. The digested sample was purified by Sep-Pak C18 Classic Cartridge (Waters), collecting both the aqueous fraction containing released terminal fragments and the organic fraction containing glycopeptides. In this way, the *N*-glycan core structure remained attached to the hydrophobic peptide and could be completely removed, and the hydrophilic termini could be isolated. The released termini were permethylated following the method for *O-*glycans and analyzed by MALDI-TOF-TOF (36, 60, 61). MS/MS fragments were annotated by manually calculating all the possible fragmentations from the hypothesized glycans based on composition and based on the polylactosamine template Galβ1-4GlcNAcβ1–3Gal-itol and then matching with observed diagnostic fragments. All known cross-ring fragmentations and co-elimination of glycan-ring substituents were included in the calculation (64, 65).

### RT-qPCR

SKOV3 total RNA was extracted using the RNeasy mini kit column (Qiagen) following the manufacturer's instructions, including a 15-min DNase (Qiagen) digestion at room temperature to remove genomic DNA. RT-qPCR was performed using SYBR Green (Thermo Fisher Scientific) for 40 cycles on a 7500 Fast Real Time PCR System (Applied Biosystems). The primers used are listed in supplemental Table 4. Levels of starting mRNA were calculated using the LinregPCR method and software (66, 67). Gene expression was normalized to GAPDH because normalizing to the geometric mean of three housekeeping genes GAPDH, RPS13, and SDHA showed no significant difference compared with normalizing to only GAPDH (data not shown) (68).

### Meta-analysis of gene expression

Data for SKOV3 expression of GAPDH, FUT1, FUT2, B3GALT1, and B3GALT5 were obtained from the CCLE through the BioGPS web portal (39). B3GALT2 data were unavailable for SKOV3 in this dataset.

### siRNA knockdown

SKOV3 was reverse-transfected with siRNA targeting three sites on exon 3 of B3GALT5 (Silencer Select, Life Technologies, Inc.) with Lipofectamine (Life Technologies, Inc.), following the manufacturer's instructions. Exon 3 on B3GALT5 was targeted because it was common across the known variants. SKOV3 cells were seeded with final siRNA concentrations of 0, 36, 60, or 120 nm for 72 h. The siRNA sequences are listed in supplemental Table 5.

## Author contributions

M. C. prepared the manuscript, performed, and analyzed the data from the cell culture, mass spectrometry, and siRNA. B. L. and L. H. performed the glycan microarrays and analyzed the data. A. C., V. D., and H. L. T. generated mAb-A4 and performed cell line flow cytometry screening and immunohistochemistry. M. C., R. C., and O. B. generated and analyzed MALDI-QIT-TOF data. A. C., A. D., and S. M. H. conceived of and coordinated the study. All authors edited the paper.

## Supplementary Material

Supplemental Data

## Footnotes

4 The abbreviations used are:

OC: ovarian cancer
hESC: human embryonic stem cell
PNPA: *p*-nitrophenyl anthranilate
BisTris: 2-[bis(2-hydroxyethyl)amino]-2-(hydroxymethyl)propane-1,3-diol
IP: immunoprecipitation
LacNAc: *N*-acetyllactosamine
PNGaseF: peptide:*N*-glycosidase F
PI: propidium iodide
qPCR: quantitative PCR
CCLE: Cancer Cell Line Encyclopedia
CID: collision-induced dissociation
QIT: quadrupole-ion-trap.

## References

[1] Bowtell D. D., Böhm S., Ahmed A. A., Aspuria P.-J., Bast R. C. Jr., Beral V., Berek J. S., Birrer M. J., Blagden S., Bookman M. A., Brenton J. D., Chiappinelli K. B., Martins F. C., Coukos G., Drapkin R., et al (2015) Rethinking ovarian cancer II: reducing mortality from high-grade serous ovarian cancer. Nat. Rev. Cancer 15, 668–679 26493647 10.1038/nrc4019 PMC4892184

[2] Coward J. I., Middleton K., and Murphy F. (2015) New perspectives on targeted therapy in ovarian cancer. Int. J. Womens Health 7, 189–203 25678824 10.2147/IJWH.S52379 PMC4324539

[3] Katsumata N., Yasuda M., Isonishi S., Takahashi F., Michimae H., Kimura E., Aoki D., Jobo T., Kodama S., Terauchi F., Sugiyama T., Ochiai K., and Japanese Gynecologic Oncology Group. (2013) Long-term results of dose-dense paclitaxel and carboplatin *versus* conventional paclitaxel and carboplatin for treatment of advanced epithelial ovarian, fallopian tube, or primary peritoneal cancer (JGOG 3016): a randomised, controlled, open-label trial. Lancet Oncol. 14, 1020–1026 23948349 10.1016/S1470-2045(13)70363-2

[4] Kemp Z., and Ledermann J. (2013) Update on first-line treatment of advanced ovarian carcinoma. Int. J. Womens Health 5, 45–51 23378788 10.2147/IJWH.S30231 PMC3558307

[5] McGuire W. P., Hoskins W. J., Brady M. F., Kucera P. R., Partridge E. E., Look K. Y., Clarke-Pearson D. L., and Davidson M. (1996) Cyclophosphamide and cisplatin compared with paclitaxel and cisplatin in patients with stage III and stage IV ovarian cancer. N. Engl. J. Med. 334, 1–6 7494563 10.1056/NEJM199601043340101

[6] National Cancer Institute (2015) Ovarian epithelial, fallopian tube, primary peritoneal cancer. Natl. Cancer Inst. www.cancer.gov/types/ovarian/hp/ovarian-epithelial-treatment-pdq#section/_82 (Accessed November 30, 2015) 26389163

[7] Abbott K. L., Lim J.-M., Wells L., Benigno B. B., McDonald J. F., and Pierce M. (2010) Identification of candidate biomarkers with cancer-specific glycosylation in the tissue and serum of endometrioid ovarian cancer patients by glycoproteomic analysis. Proteomics 10, 470–481 19953551 10.1002/pmic.200900537 PMC4932840

[8] Abbott K. L., Nairn A. V., Hall E. M., Horton M. B., McDonald J. F., Moremen K. W., Dinulescu D. M., and Pierce M. (2008) Focused glycomic analysis of the *N*-linked glycan biosynthetic pathway in ovarian cancer. Proteomics 8, 3210–3220 18690643 10.1002/pmic.200800157 PMC3970323

[9] Anugraham M., Jacob F., Nixdorf S., Everest-Dass A. V., Heinzelmann-Schwarz V., and Packer N. H. (2014) Specific glycosylation of membrane proteins in epithelial ovarian cancer cell lines: glycan structures reflect gene expression and DNA methylation status. Mol. Cell. Proteomics 13, 2213–2232 24855066 10.1074/mcp.M113.037085 PMC4159645

[10] Machado E., Kandzia S., Carilho R., Altevogt P., Conradt H. S., and Costa J. (2011) *N*-Glycosylation of total cellular glycoproteins from the human ovarian carcinoma SKOV3 cell line and of recombinantly expressed human erythropoietin. Glycobiology 21, 376–386 21030537 10.1093/glycob/cwq170

[11] Kui Wong N., Easton R. L., Panico M., Sutton-Smith M., Morrison J. C., Lattanzio F. A., Morris H. R., Clark G. F., Dell A., and Patankar M. S. (2003) Characterization of the oligosaccharides associated with the human ovarian tumor marker CA125. J. Biol. Chem. 278, 28619–28634 12734200 10.1074/jbc.M302741200

[12] Ricardo S., Marcos-Silva L., Pereira D., Pinto R., Almeida R., Söderberg O., Mandel U., Clausen H., Felix A., Lunet N., and David L. (2015) Detection of glyco-mucin profiles improves specificity of MUC16 and MUC1 biomarkers in ovarian serous tumours. Mol. Oncol. 9, 503–512 25454345 10.1016/j.molonc.2014.10.005 PMC5528651

[13] Gao L., Yan L., Lin B., Gao J., Liang X., Wang Y., Liu J., Zhang S., and Iwamori M. (2011) Enhancive effects of Lewis y antigen on CD44-mediated adhesion and spreading of human ovarian cancer cell line RMG-I. J. Exp. Clin. Cancer Res. 30, 15 21294926 10.1186/1756-9966-30-15 PMC3045975

[14] Pochechueva T., Jacob F., Fedier A., and Heinzelmann-Schwarz V. (2012) Tumor-associated glycans and their role in gynecological cancers: accelerating translational research by novel high-throughput approaches. Metabolites 2, 913–939 24957768 10.3390/metabo2040913 PMC3901231

[15] Wang Y., Liu J., Lin B., Wang C., Li Q., Liu S., Yan L., Zhang S., and Iwamori M. (2011) Study on the expression and clinical significances of Lewis y antigen and integrin αv, β3 in epithelial ovarian tumors. Int. J. Mol. Sci. 12, 3409–3421 21747684 10.3390/ijms12063409 PMC3131568

[16] Hakomori S. (2001) Tumor-associated carbohydrate antigens defining tumor malignancy: basis for development of anti-cancer vaccines. Adv. Exp. Med. Biol. 491, 369–402 14533809 10.1007/978-1-4615-1267-7_24

[17] Miles D., Roché H., Martin M., Perren T. J., Cameron D. A., Glaspy J., Dodwell D., Parker J., Mayordomo J., Tres A., Murray J. L., Ibrahim N. K., and Theratope® Study Group. (2011) Phase III multicenter clinical trial of the sialyl-TN (STn)-keyhole limpet hemocyanin (KLH) vaccine for metastatic breast cancer. Oncologist 16, 1092–1100 21572124 10.1634/theoncologist.2010-0307 PMC3228158

[18] O'Cearbhaill R. E., Iasonos A., Ragupathi G., Danishefsky S., and Sabbatini P. (2014) Unimolecular pentavalent (globo-H-Gm2-Stn-Tf-Tn) immunization of patients (pts) with epithelial ovarian(eoc), fallopian tube, or peritoneal cancer in first remission. Ann. Oncol. 25, iv311 10.3390/cancers8040046 PMC4846855 27110823

[19] Lucena M. C., Carvalho-Cruz P., Donadio J. L., Oliveira I. A., de Queiroz R. M., Marinho-Carvalho M. M., Sola-Penna M., de Paula I. F., Gondim K. C., McComb M. E., Costello C. E., Whelan S. A., Todeschini A. R., and Dias W. B. (2016) Epithelial mesenchymal transition induces aberrant glycosylation through hexosamine biosynthetic pathway activation. J. Biol. Chem. 291, 12917–12929 27129262 10.1074/jbc.M116.729236 PMC4933211

[20] Nonaka M., Fukuda M. N., Gao C., Li Z., Zhang H., Greene M. I., Peehl D. M., Feizi T., and Fukuda M. (2014) Determination of carbohydrate structure recognized by prostate-specific F77 monoclonal antibody through expression analysis of glycosyltransferase genes. J. Biol. Chem. 289, 16478–16486 24753248 10.1074/jbc.M114.559047 PMC4047414

[21] Gao C., Liu Y., Zhang H., Zhang Y., Fukuda M. N., Palma A. S., Kozak R. P., Childs R. A., Nonaka M., Li Z., Siegel D. L., Hanfland P., Peehl D. M., Chai W., Greene M. I., and Feizi T. (2014) Carbohydrate sequence of the prostate cancer-associated antigen F77 assigned by a mucin *O-*glycome designer array. J. Biol. Chem. 289, 16462–16477 24753245 10.1074/jbc.M114.558932 PMC4047413

[22] Chua J. X., Vankemmelbeke M., McIntosh R. S., Clarke P. A., Moss R., Parsons T., Spendlove I., Zaitoun A. M., Madhusudan S., and Durrant L. G. (2015) Monoclonal antibodies targeting LecLex-related glycans with potent antitumor activity. Clin. Cancer Res. 21, 2963–2974 25779947 10.1158/1078-0432.CCR-14-3030

[23] Shibata T. K., Matsumura F., Wang P., Yu S., Chou C.-C., Khoo K.-H., Kitayama K., Akama T. O., Sugihara K., Kanayama N., Kojima-Aikawa K., Seeberger P. H., Fukuda M., Suzuki A., Aoki D., and Fukuda M. N. (2012) Identification of mono- and disulfated *N*-acetyl-lactosaminyl oligosaccharide structures as epitopes specifically recognized by humanized monoclonal antibody HMOCC-1 raised against ovarian cancer. J. Biol. Chem. 287, 6592–6602 22194598 10.1074/jbc.M111.305334 PMC3307324

[24] Suzuki N., Aoki D., Tamada Y., Susumu N., Orikawa K., Tsukazaki K., Sakayori M., Suzuki A., Fukuchi T., Mukai M., Kojima-Aikawa K., Ishida I., and Nozawa S. (2004) HMOCC-1, a human monoclonal antibody that inhibits adhesion of ovarian cancer cells to human mesothelial cells. Gynecol. Oncol. 95, 290–298 15491748 10.1016/j.ygyno.2004.06.024

[25] Loo D., Pryer N., Young P., Liang T., Coberly S., King K. L., Kang K., Roberts P., Tsao M., Xu X., Potts B., and Mather J. P. (2007) The glycotope-specific RAV12 monoclonal antibody induces oncosis *in vitro* and has antitumor activity against gastrointestinal adenocarcinoma tumor xenografts *in vivo*. Mol. Cancer Ther. 6, 856–865 17363480 10.1158/1535-7163.MCT-06-0581

[26] Coberly S. K., Chen F. Z., Armanini M. P., Chen Y., Young P. F., Mather J. P., and Loo D. T. (2009) The RAV12 monoclonal antibody recognizes the *N*-linked glycotope RAAG12: expression in human normal and tumor tissues. Arch. Pathol. Lab. Med. 133, 1403–1412 19722746 10.5858/133.9.1403

[27] Shoreibah M. G., Jackson C. L., Price P. W., Meagher R., Godwin A. K., Cai Q., and Gildersleeve J. C. (2011) Anti-human embryonic stem cell monoclonal antibody Hesca-2 binds to a glycan epitope commonly found on carcinomas. Stem Cells Dev. 20, 515–525 20887211 10.1089/scd.2010.0172 PMC3128762

[28] Ruhaak L. R., Miyamoto S., and Lebrilla C. B. (2013) Developments in the identification of glycan biomarkers for the detection of cancer. Mol. Cell. Proteomics 12, 846–855 23365456 10.1074/mcp.R112.026799 PMC3617331

[29] Choo A. B., Tan H. L., Ang S. N., Fong W. J., Chin A., Lo J., Zheng L., Hentze H., Philp R. J., Oh S. K., and Yap M. (2008) Selection against undifferentiated human embryonic stem cells by a cytotoxic antibody recognizing podocalyxin-like protein-1. Stem Cells 26, 1454–1463 18356574 10.1634/stemcells.2007-0576

[30] Tan H. L., Fong W. J., Lee E. H., Yap M., and Choo A. (2009) mAb 84, a cytotoxic antibody that kills undifferentiated human embryonic stem cells via oncosis. Stem Cells 27, 1792–1801 19544435 10.1002/stem.109

[31] Escrevente C., Machado E., Brito C., Reis C. A., Stoeck A., Runz S., Marmé A., Altevogt P., and Costa J. (2006) Different expression levels of α3/4 fucosyltransferases and Lewis determinants in ovarian carcinoma tissues and cell lines. Int. J. Oncol. 29, 557–566 16865271

[32] Fukuda M. N., and Matsumura G. (1976) Endo-β-galactosidase of *Escherichia freundii*. Purification and endoglycosidic action on keratan sulfates, oligosaccharides, and blood group active glycoprotein. J. Biol. Chem. 251, 6218–6225 135762

[33] Scudder P., Hanfland P., Uemura K., and Feizi T. (1984) Endo-β-d-galactosidases of *Bacteroides fragilis* and *Escherichia freundii* hydrolyze linear but not branched oligosaccharide domains of glycolipids of the neolacto series. J. Biol. Chem. 259, 6586–6592 6427218

[34] Dell A., Carman N. H., Tiller P. R., and Thomas-Oates J. E. (1988) Fast atom bombardment mass spectrometric strategies for characterizing carbohydrate-containing biopolymers. Biol. Mass Spectrom. 16, 19–24 10.1002/bms.1200160104 3242669

[35] Ismail M. N., Stone E. L., Panico M., Lee S. H., Luu Y., Ramirez K., Ho S. B., Fukuda M., Marth J. D., Haslam S. M., and Dell A. (2011) High-sensitivity *O-*glycomic analysis of mice deficient in core 2 1,6-*N*-acetylglucosaminyltransferases. Glycobiology 21, 82–98 20855471 10.1093/glycob/cwq134 PMC2998984

[36] Dell A., Reason A. J., Khoo K.-H., Panico M., McDowell R. A., and Morris H. R. (1994) Mass spectrometry of carbohydrate-containing biopolymers. Methods Enzymol. 230, 108–132 8139492 10.1016/0076-6879(94)30010-0

[37] Huang R. Y., Wong M. K., Tan T. Z., Kuay K. T., Ng A. H., Chung V. Y., Chu Y.-S., Matsumura N., Lai H.-C., Lee Y. F., Sim W.-J., Chai C., Pietschmann E., Mori S., Low J. J., Choolani M., and Thiery J. P. (2013) An EMT spectrum defines an anoikis-resistant and spheroidogenic intermediate mesenchymal state that is sensitive to E-cadherin restoration by an src-kinase inhibitor, saracatinib (AZD0530). Cell Death Dis. 4, e915 24201814 10.1038/cddis.2013.442 PMC3847320

[38] Tan T. Z., Miow Q. H., Huang R. Y.-J., Wong M. K., Ye J., Lau J. A., Wu M. C., Bin Abdul Hadi L. H., Soong R., Choolani M., Davidson B., Nesland J. M., Wang L.-Z., Matsumura N., Mandai M., Konishi I., Goh B.-C., Chang J. T., Thiery J. P., and Mori S. (2013) Functional genomics identifies five distinct molecular subtypes with clinical relevance and pathways for growth control in epithelial ovarian cancer. EMBO Mol. Med. 5, 983–998 10.1002/emmm.201201823 PMC3721473 23666744

[39] Barretina J., Caponigro G., Stransky N., Venkatesan K., Margolin A. A., Kim S., Wilson C. J., Lehár J., Kryukov G. V., Sonkin D., Reddy A., Liu M., Murray L., Berger M. F., Monahan J. E., et al (2012) The cancer cell line encyclopedia enables predictive modelling of anticancer drug sensitivity. Nature 483, 603–607 22460905 10.1038/nature11003 PMC3320027

[40] Tang C., Lee A. S., Volkmer J.-P., Sahoo D., Nag D., Mosley A. R., Inlay M. A., Ardehali R., Chavez S. L., Pera R. R., Behr B., Wu J. C., Weissman I. L., and Drukker M. (2011) An antibody against SSEA-5 glycan on human pluripotent stem cells enables removal of teratoma-forming cells. Nat. Biotechnol. 29, 829–834 21841799 10.1038/nbt.1947 PMC3537836

[41] Natunen S., Satomaa T., Pitkänen V., Salo H., Mikkola M., Natunen J., Otonkoski T., and Valmu L. (2011) The binding specificity of the marker antibodies Tra-1–60 and Tra-1–81 reveals a novel pluripotency-associated type 1 lactosamine epitope. Glycobiology 21, 1125–1130 21159783 10.1093/glycob/cwq209 PMC3150112

[42] Satomaa T., Heiskanen A., Mikkola M., Olsson C., Blomqvist M., Tiittanen M., Jaatinen T., Aitio O., Olonen A., Helin J., Hiltunen J., Natunen J., Tuuri T., Otonkoski T., Saarinen J., and Laine J. (2009) The *N*-glycome of human embryonic stem cells. BMC Cell Biol. 10, 42 19490625 10.1186/1471-2121-10-42 PMC2696424

[43] Tateno H., Toyota M., Saito S., Onuma Y., Ito Y., Hiemori K., Fukumura M., Matsushima A., Nakanishi M., Ohnuma K., Akutsu H., Umezawa A., Horimoto K., Hirabayashi J., and Asashima M. (2011) Glycome diagnosis of human induced pluripotent stem cells using lectin microarray. J. Biol. Chem. 286, 20345–20353 21471226 10.1074/jbc.M111.231274 PMC3121447

[44] Liang Y.-J., Kuo H.-H., Lin C.-H., Chen Y.-Y., Yang B.-C., Cheng Y.-Y., Yu A. L., Khoo K.-H., and Yu J. (2010) Switching of the core structures of glycosphingolipids from globo- and lacto- to ganglio-series upon human embryonic stem cell differentiation. Proc. Natl. Acad. Sci. 107, 22564–22569 21149694 10.1073/pnas.1007290108 PMC3012484

[45] Liang Y.-J., Yang B.-C., Chen J.-M., Lin Y.-H., Huang C.-L., Cheng Y.-Y., Hsu C.-Y., Khoo K.-H., Shen C.-N., and Yu J. (2011) Changes in glycosphingolipid composition during differentiation of human embryonic stem cells to ectodermal or endodermal lineages. Stem Cells 29, 1995–2004 21956927 10.1002/stem.750

[46] Ojima T., Shibata E., Saito S., Toyoda M., Nakajima H., Yamazaki-Inoue M., Miyagawa Y., Kiyokawa N., Fujimoto J., Sato T., and Umezawa A. (2015) Glycolipid dynamics in generation and differentiation of induced pluripotent stem cells. Sci. Rep. 5, 14988 26477663 10.1038/srep14988 PMC4609952

[47] Uhlén M., Fagerberg L., Hallström B. M., Lindskog C., Oksvold P., Mardinoglu A., Sivertsson Å., Kampf C., Sjöstedt E., Asplund A., Olsson I., Edlund K., Lundberg E., Navani S., Szigyarto C. A., et al (2015) Proteomics. Tissue-based map of the human proteome. Science 347, 1260419 25613900 10.1126/science.1260419

[48] Isshiki S., Togayachi A., Kudo T., Nishihara S., Watanabe M., Kubota T., Kitajima M., Shiraishi N., Sasaki K., Andoh T., and Narimatsu H. (1999) Cloning, expression, and characterization of a novel UDP-galactose:β-*N*-acetylglucosamine β1,3-galactosyltransferase (β3Gal-T5) responsible for synthesis of type 1 chain in colorectal and pancreatic epithelia and tumor cells derived there from. J. Biol. Chem. 274, 12499–12507 10212226 10.1074/jbc.274.18.12499

[49] Zulueta A. (2014) Transcriptional Regulation of the B3GALT5 Gene. Doctoral Thesis, Università degli Studi di Milano, Milan, Italy

[50] Trinchera M., Zulueta A., Caretti A., and Dall'Olio F. (2014) Control of glycosylation-related genes by DNA methylation: the intriguing case of the B3GALT5 gene and its distinct promoters. Biology 3, 484–497 25256425 10.3390/biology3030484 PMC4192623

[51] Reya T., Morrison S. J., Clarke M. F., and Weissman I. L. (2001) Stem cells, cancer, and cancer stem cells. Nature 414, 105–111 11689955 10.1038/35102167

[52] Reya T., and Clevers H. (2005) Wnt signalling in stem cells and cancer. Nature 434, 843–850 15829953 10.1038/nature03319

[53] Ben-Porath I., Thomson M. W., Carey V. J., Ge R., Bell G. W., Regev A., and Weinberg R. A. (2008) An embryonic stem cell-like gene expression signature in poorly differentiated aggressive human tumors. Nat. Genet. 40, 499–507 18443585 10.1038/ng.127 PMC2912221

[54] Polyak K., and Weinberg R. A. (2009) Transitions between epithelial and mesenchymal states: acquisition of malignant and stem cell traits. Nat. Rev. Cancer 9, 265–273 19262571 10.1038/nrc2620

[55] Lee J., Gan H. T., Latiff S. M., Chuah C., Lee W. Y., Yang Y.-S., Loo B., Ng S. K., and Gagnon P. (2012) Principles and applications of steric exclusion chromatography. J. Chromatogr. A 1270, 162–170 23182281 10.1016/j.chroma.2012.10.062

[56] Kostlánová N., Mitchell E. P., Lortat-Jacob H., Oscarson S., Lahmann M., Gilboa-Garber N., Chambat G., Wimmerová M., and Imberty A. (2005) The fucose-binding lectin from *Ralstonia solanacearum*. A new type of β-propeller architecture formed by oligomerization and interacting with fucoside, fucosyllactose, and plant xyloglucan. J. Biol. Chem. 280, 27839–27849 15923179 10.1074/jbc.M505184200

[57] Duk M., Ugorski M., and Lisowska E. (1997) beta-elimination of *O-*glycans from glycoproteins transferred to immobilon P membranes: method and some applications. Anal. Biochem. 253, 98–102 9356147 10.1006/abio.1997.9994

[58] Day C. J., Tiralongo J., Hartnell R. D., Logue C.-A., Wilson J. C., von Itzstein M., and Korolik V. (2009) Differential carbohydrate recognition by *Campylobacter jejuni* strain 11168: influences of temperature and growth conditions. PLOS ONE. 4, e4927 19290056 10.1371/journal.pone.0004927 PMC2654152

[59] Waespy M., Gbem T. T., Elenschneider L., Jeck A.-P., Day C. J., Hartley-Tassell L., Bovin N., Tiralongo J., Haselhorst T., and Kelm S. (2015) Carbohydrate recognition specificity of trans-sialidase lectin domain from trypanosoma congolense. PLoS Negl. Trop. Dis. 9, e0004344 26474304 10.1371/journal.pntd.0004120 PMC4608562

[60] Sutton-Smith M., and Dell A. (2006) Analysis of carbohydrates/glycoproteins by mass spectrometry. Cell Biol. Lab. Handb. 4, 425–425

[61] Haslam S. M., North S. J., and Dell A. (2006) Mass spectrometric analysis of *N*- and *O-*glycosylation of tissues and cells. Curr. Opin. Struct. Biol. 16, 584–591 16938453 10.1016/j.sbi.2006.08.006

[62] Ciucanu I., and Kerek F. (1984) A simple and rapid method for the permethylation of carbohydrates. Carbohydr. Res. 131, 209–217

[63] Ceroni A., Maass K., Geyer H., Geyer R., Dell A., and Haslam S. M. (2008) GlycoWorkbench: a tool for the computer-assisted annotation of mass spectra of glycans. J. Proteome Res. 7, 1650–1659 18311910 10.1021/pr7008252

[64] Domon B., and Costello C. E. (1988) A systematic nomenclature for carbohydrate fragmentations in FAB-MS/MS spectra of glycoconjugates. Glycoconj. J. 5, 397–409

[65] Spina E., Sturiale L., Romeo D., Impallomeni G., Garozzo D., Waidelich D., and Glueckmann M. (2004) New fragmentation mechanisms in matrix-assisted laser desorption/ionization time-of-flight/time-of-flight tandem mass spectrometry of carbohydrates. Rapid Commun. Mass Spectrom. 18, 392–398 14966845 10.1002/rcm.1350

[66] Ruijter J. M., Pfaffl M. W., Zhao S., Spiess A. N., Boggy G., Blom J., Rutledge R. G., Sisti D., Lievens A., De Preter K., Derveaux S., Hellemans J., and Vandesompele J. (2013) Evaluation of qPCR curve analysis methods for reliable biomarker discovery: bias, resolution, precision, and implications. Methods 59, 32–46 22975077 10.1016/j.ymeth.2012.08.011

[67] Ruijter J. M., Lorenz P., Tuomi J. M., Hecker M., and van den Hoff M. J. (2014) Fluorescent-increase kinetics of different fluorescent reporters used for qPCR depend on monitoring chemistry, targeted sequence, type of DNA input and PCR efficiency. Mikrochim. Acta 181, 1689–1696 25253910 10.1007/s00604-013-1155-8 PMC4167442

[68] Jacob F., Guertler R., Naim S., Nixdorf S., Fedier A., Hacker N. F., and Heinzelmann-Schwarz V. (2013) Careful selection of reference genes is required for reliable performance of RT-qPCR in human normal and cancer cell lines. PLoS ONE 8, e59180 23554992 10.1371/journal.pone.0059180 PMC3598660

