# Supplementary material for: Characterization of H type 1 and type 1 N-acetyllactosamine glycan epitopes on ovarian cancer specifically recognized by the anti-glycan monoclonal antibody mAb-A4
Source: J Biol Chem. 2017 Feb 6;292(15):6163–76. doi: 10.1074/jbc.M116.768887 (PMC5391748; doi:10.1074/jbc.M116.768887)
Supplement: Supplemental Data [file supp_292_15_6163__index.html]

Characterization of H type 1 and type 1 N-acetyllactosamine glycan epitopes on ovarian cancer specifically recognized by the anti-glycan monoclonal antibody mAb-A4 — Characterization of H type 1 and type 1 N-acetyllactosamine glycan epitopes on ovarian cancer specifically recognized by the anti-glycan monoclonal antibody mAb-A4 — Ovarian cancer glycan epitopes recognized by mAb-A4 — Supplemental Data 

# Characterization of H type 1 and type 1 *N*-acetyllactosamine glycan epitopes on ovarian cancer specifically recognized by the anti-glycan monoclonal antibody mAb-A4

## Supplemental Data

- Supplementary information (.pdf, 951 KB)
- Supplementary table - list of glycans on microarray (.xlsx, 32 KB) - List of glycans used in the glycan microarray
