# Supplementary material for: Characterization of H type 1 and type 1 N-acetyllactosamine glycan epitopes on ovarian cancer specifically recognized by the anti-glycan monoclonal antibody mAb-A4
Source: J Biol Chem. 2017 Feb 6;292(15):6163–76. doi: 10.1074/jbc.M116.768887 (PMC5391748; doi:10.1074/jbc.M116.768887)
Supplement: Supplemental Data [file 10.1074_M116.768887_jbc.M116.768887-1.pdf]

Characterization of H Type 1 and Type 1 N-acetyllactosamine Glycan Epitopes on Ovarian Cancer Specifically Recognized by the Anti-Glycan Monoclonal Antibody mAb-A4

Matthew S. F. Choo[†§], Heng Liang Tan[†], Vanessa Ding[†], Roberto Castangia[¤], Omar Belgacem[¤], Brian Liau[†], Lauren Hartley-Tassell[‡], Stuart M. Haslam[§], Anne Dell[§], Andre B. H. Choo[†]

From the [§]Department of Life Sciences, Imperial College London, SW7 2AZ, UK, the [†]Bioprocessing Technology Institute, Singapore 138668, the [‡]Institute for Glycomics, Griffith University, Southport, Queensland, Australia 4215, and [¤]Shimadzu, Manchester, UK.

SUPPLEMENTARY TABLES AND FIGURES

SUPPLEMENTARY TABLES

Supplementary Table 1

Supplementary Table 1. Glycan standards used in this work.

Glycan Structure	Name	Source
Galβ1-3(Fucα1-4)GlcNAc	Lewis A, LeA	Dextra Laboratories, UK
Fucα1-2Galβ1-3(Fucα1-4)GlcNAc	Lewis B, LeB	
Galβ1-4(Fucα1-3)GlcNAc	Lewis X, LeX	
Fucα1-2Galβ1-4(Fucα1-3)GlcNAc	Lewis Y, LeY	
GalNAcα1-3Fucα1-2Gal	Blood Group A, bgA	
Galα1-3Fucα1-2Gal	Blood Group B, bgB	
Fucα1-2Galβ1-3GlcNAc	H Type 1 , H1	
Fucα1-2Galβ1-4GlcNAc	H Type 2, H2	
Fucα1-2Galβ1-3GlcNAcβ1-3Galβ1-4Glc	Lacto-N-fucopentaose I, LNFP1	
Fucα1-2Galβ1-3GlcNAcβ1-3Galβ1-4Glc	Lacto-N-fucopentaose II LNFP2	
Galβ1-3GlcNAcβ1-3Galβ1-4Glc	Type 1 tetrasaccharide	Oligotech-Elicityl, France
Fucα1-2Galβ1-4GlcNAcβ1-3Galβ1-4Glc	Lacto-N-neofucopentaose I, LNnFP1	

Supplementary Table 2

Supplementary Table 2. Properties of ovarian cancer and normal cell lines used in this study. The known subtype and EMT classification was compared against mAb-A4 binding on FACS, western blot and sensitivity to PNGaseF and endo-β-galactosidase.

Cell Line	Subtyping*	EMT^	mAb-A4 FACS binding §	mAb-A4 western blot ¤	PNGaseF sensitive?†	Endo-β-gal sensitive?‡
SKOV3	Mesenchymal	IM	+	+	+	+
IGROV1	Stem-A1	IE	+	+	+	+
OV90	Stem-B	E	+	+	Partial	Partial
OVCA433	Epi-A	E	+	+	Partial	Partial
HEY A8	Mesenchymal	M	+	-	nd	nd
OVCAR-3	Stem-A	E	+	-	nd	nd
OVCAR8	Epithelial-A	E	-	-	nd	nd
IOSE523	N/A	N/A	-	-	nd	nd

*Molecular subtyping by Tan et al., 2013 (1).
^EMT phenotypic typing by Huang et al., 2013; E=epithelial, IE=intermediate epithelial, IM=intermediate mesenchymal, M=mesenchymal.
§ a plus represents positive FACS binding of mAb-A4 and a minus indicates no FACS binding.
¤ a plus indicates that western blot of that cell lysate was positive for mAb-A4 binding and a minus indicates lack of mAb-A4 binding signal.
†Western blot signal of mAb-A4 in that cell line was sensitive to PNGaseF digestion.
‡Western blot signal of mAb-A4 in that cell line was sensitive to endo-β-galactosidase digestion.
nd, not done due to lack of normal western blot signal.

Supplementary Table 3

Supplementary Table 3. Diagnostic ions of possible isomers in the *m/z* 913 precursor, and their mathematical derivations. Fragmentation nomenclature was adapted from Domon and Costello (2), modified by Spina et al. (3).

Isomer	*m/z*	Assigned Ion
LeX	707	(913-14-174-18=707) Z ion; Elimination of Fuc from FucGalGlcNAcGal.
	633	(707-74=633) $Z^{0,4}X$ ion; Cross-ring cleavage plus elimination of Fuc from FucGalGlcNAcGal.
	472	(660+18-14-174-18=472) D ion; Elimination of Fuc from FucGalGlcNAc+H2O
	454	(660-14-174-18=454) D ion - H2O; Elimination of Fuc from FucGalGlcNAc
	329	3,5A Cross-ring cleavage of GlcNAc unique to LeX.
LeA	677	(913-14-204-18=677) Z ion; Elimination of Gal from FucGalGlcNAcGal.
	603	(677-74=603) $Z^{0,4}X$ ion; Cross-ring cleavage plus elimination of Gal from FucGalGlcNAcGal.
	442	(660+18-14-204-18=472) D ion; Elimination of Gal from FucGalGlcNAc+H2O.
H2	646	(660+18-32=646) D ion; Elimination of methanol from FucGalGlcNAc+H2O.
	628	(660-32=628) D ion; Elimination of methanol from FucGalGlcNAc.
H1	503	(913-14-204-174-18=503) Z ion; Elimination of FucGal from FucGalGlcNAcGal.
	521	(913-14-204-174=521) Y ion; Cleavage of FucGal from FucGalGlcNAcGal.
	429	(503-74=429) $Z^{0,4}X$ ion; Cross-ring cleavage plus elimination of FucGal from FucGalGlcNAcGal.

Supplementary Table 4

Supplementary Table 4. Primers used for RT-qPCR. Primers were based on literature (4) and were purchased from iDNA Biotechnology (Singapore).

Gene	Forward	Reverse
GAPDH	GTCGGAGTCAACGGATTTGG	AAAAGCAGCCCTGGTGACC
FUT1	GCAGGCCATGGACTGGTT	CCTGGGAGGTGTCGATGTTT
FUT2	CTCGCTACAGCTCCCTCATCTT	CGTGGGAGGTGTCAATGTTCT
B3GALT1	TGGAAAATGGCCTACAGTTTGTG	GATTCTGTGCATTTCTTCTGGAGAG
B3GALT2	TTACCTCTCATCAGTTCCAGCCTAG	TGGCACAGGCATTGTGCTTA
B3GALT5	TGTCTCCAAGAGCGTCCCA	AAGAGGCATACGGAGAAGCG

Supplementary Table 5

Supplementary Table 5. siRNA sequences (Life Technologies) used for knockdown of B3GALT5.

**Product code**	**Forward**	**Reverse**
S20170	GCAAGUGGUUUGUCAGUAAtt	UUACUGACAAACCACUUGCtg
S20171	AGACCAUGAUGGGCAUAGAtt	AGACCAUGAUGGGCAUAGAtt
S20172	AGUCCUUUGUUUACAAGAAtt	UUCUUGUAAACAAAGGACUgt

Supplementary Table 6

Supplementary Table 6. **List of proteins identified by LC-ESI-MS from mAb-A4 immunopurified antigens from SKOV3 and IGROV1, indicating overlapping proteins**. SDS-PAGE bands corresponding to Western blot bands were excised and proteins were identified by shotgun proteomics.

SKOV3 (15)	OVERLAP (10)	IGROV1 (6)
Neutral alpha-glucosidase AB	Podocalyxin	Sodium-dependent phosphate transport protein 2B
Semenogelin-2	Heat shock protein HSP 90-beta	Albumin
ATP synthase subunit beta, mitochondrial	4F2 cell-surface antigen heavy chain/CD98hc	Neural cell adhesion molecule L1
Serpin H1	Lysosome-associated membrane glycoprotein 1	Endoplasmin
Probable lysosomal cobalamin transporter	Basigin/CD147	Prolactin-inducible protein
Methionine aminopeptidase 2	Desmoglein-1	Vimentin
HLA class I histocompatibility antigen, A-68 alpha chain	Neutral amino acid transporter B(0)	
CD44 antigen	Desmocollin-1	
Serum albumin	Sodium-coupled neutral amino acid transporter 2	
Zinc transporter 1	Junction plakoglobin	
High affinity cationic amino acid transporter 1		
Dynamin-1-like protein		
Neuroplastin		
Prostatic acid phosphatase		
Sodium/potassium-transporting ATPase subunit beta-1		

SUPPLEMENTARY FIGURE LEGENDS

Supplementary Figure 1. Immunohistochemistry of mAb-A4 against normal and tumor tissue. Formalin-fixed, paraffin-embedded (FFPE) tissue (Biochain, CA, USA) were stained with mAb-A4 and with secondary antibody conjugated to horseradish peroxidase, then visualised with peroxide/DAB. The tissue core shown was representative of 1-3 samples.

Supplementary Figure 2. Endo-β-galactosidase digestion of SKOV3 N-glycome. A, enzymatic specificity of endo-β-galactosidase as described earlier and observed in this work (5, 6). Brackets indicate tolerated modifications and crossed brackets indicate non-tolerated modifications. Arrows indicate digestions sites. B, comparison of MALDI-TOF spectra before and after endo-β-galactosidase treatment. SKOV3 N-glycans were digested for 48 hours with endo-β-galactosidase, permethylated and analysed by MALDI-TOF. Red crosses indicate peaks that disappeared, and blue ticks indicate peaks that became apparent or were greatly increased, relative to the reference peak at *m/z* 4226.

Supplementary Figure 3. The N-glycome of SKOV3. N-glycans were permethylated and analysed by MALDI-TOF as sodiated adducts. Annotation of peaks was by compositional analysis and fragment analysis after collisional-induced dissociation MS/MS of the peaks of interest. Asterisks (*) indicate glycans potentially carrying H Type 1.

Supplementary Figure 4. Sensitivity to enzymes of mAb-A4 epitopes from ovarian cancer cell lines. A, whole cell lysate of the indicated cell lines was western blotted and probed with mAb-A4. B, before immunoblotting with mAb-A4, whole cell lysate from SKOV3, IGROV1, OV90 and OVCA433 was digested with sialidase first followed by either no-enzyme control (“C”), PNGaseF (“P”) or endo-β-galactosidase from *E. freundii* (“E”). The size (kDa) of the lane markers is shown.

Supplementary Figure 5. MALDI-QIT-TOF MS3 analysis of the FucGalGlcNAc fragment from desialylated short N-glycans from SKOV3. Fragments of interest were isolated by the quadrupole ion trap and fragmented by collisional-induced dissociation in the trap. Partial mass spectra extending upwards were from the sample and spectra extending downwards were from a generated library: H Type 1 from LNFP1 (left), Lewis A from LNFP2 (center) and Lewis X from mouse kidney (right). A, the MS3 of the m/z 660 daughter ion from the m/z 2592 precursor. B, the MS3 of the m/z 660 daughter ion from the m/z 2418 precursor. C, for standards and samples, the ratios of *m/z* 454 and 472 peaks (by intensity) were calculated. Ticks indicate the presence of diagnostic ions m/z 433 and 329 and crosses indicate their absence. D, fragments observed from each isomer.

Supplementary Figure 6. Comparison of mAb-A4 and mAb anti-SSEA-5 binding to ovarian cancer, normal and human embryonic stem cell lines. Cell lines were harvested as single cell suspensions and were analysed by FACS with either mAb-A4 or mAb anti-SSEA-5 primary antibodies followed by anti-mouse secondary antibodies. Binding of both mAbs to HES-3 was the positive control. Shaded plots were untreated negative controls and the solid lines were antibody-treated samples. Histogram counts in each experiment were scaled to the negative control peak height.

Supplementary Figure 7. Proposed workflow for characterization of anti-glycan mAbs. The final aim is to provide both structural and functional information about the glycan epitope targeted by the mAb. The phases may occur in parallel.

Supplementary Figure 1

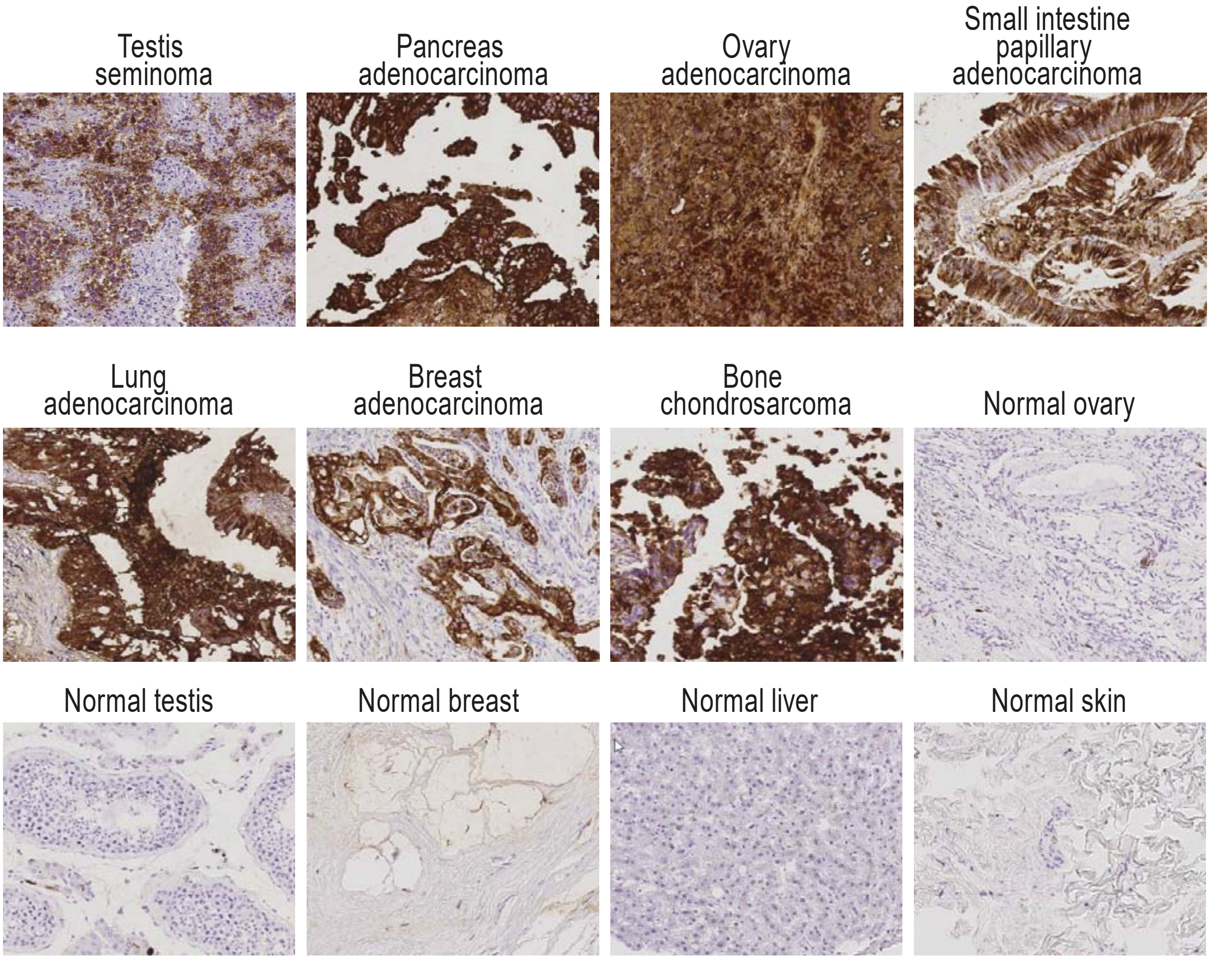

Supplementary Figure 2

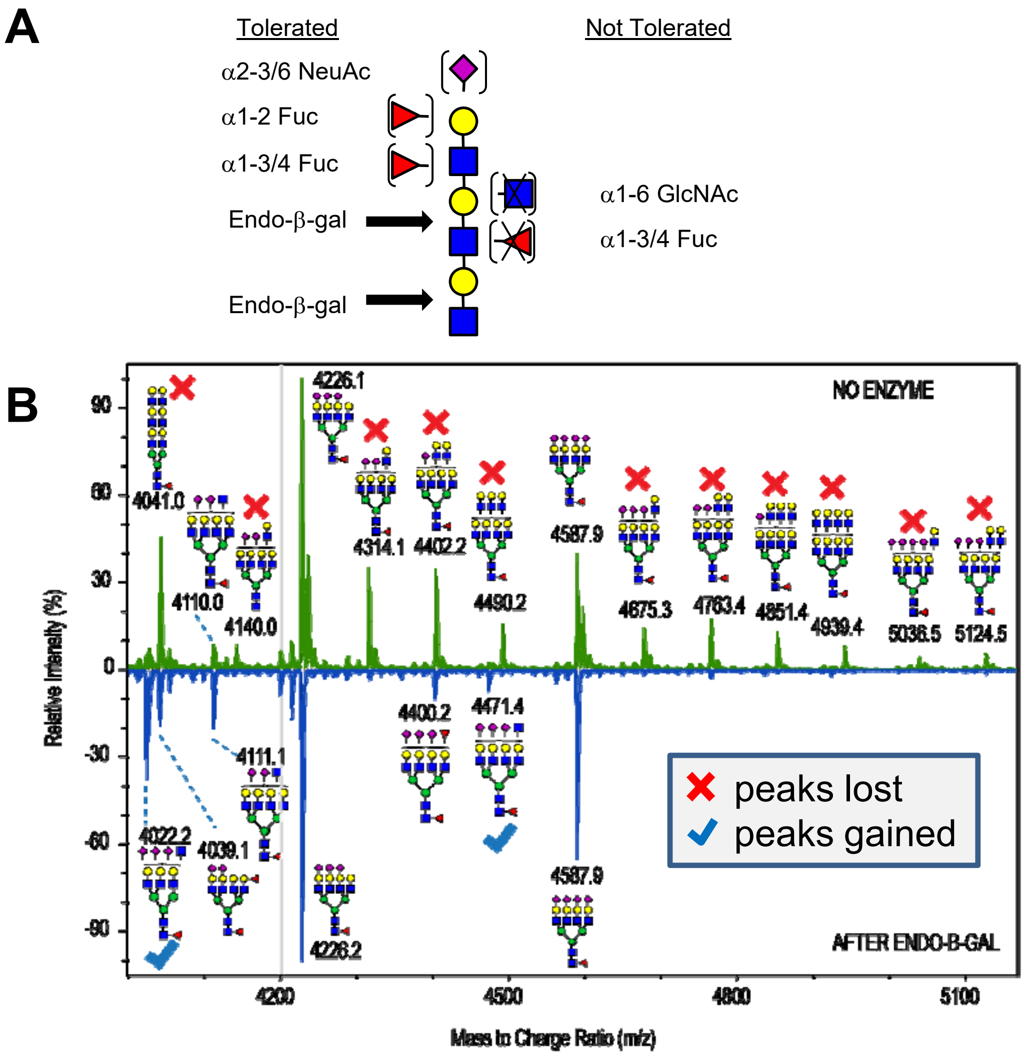

Supplementary Figure 3

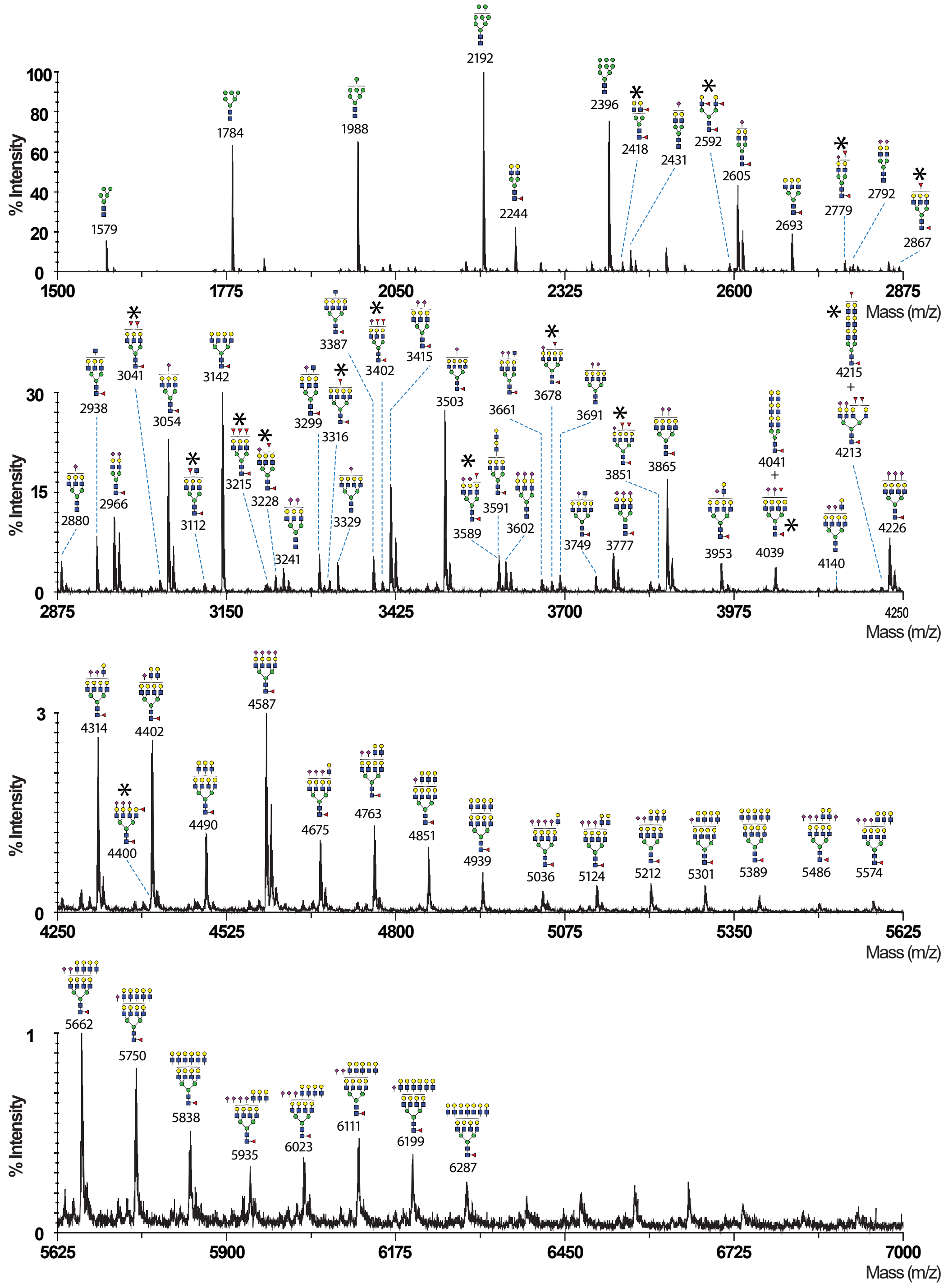

Supplementary Figure 4

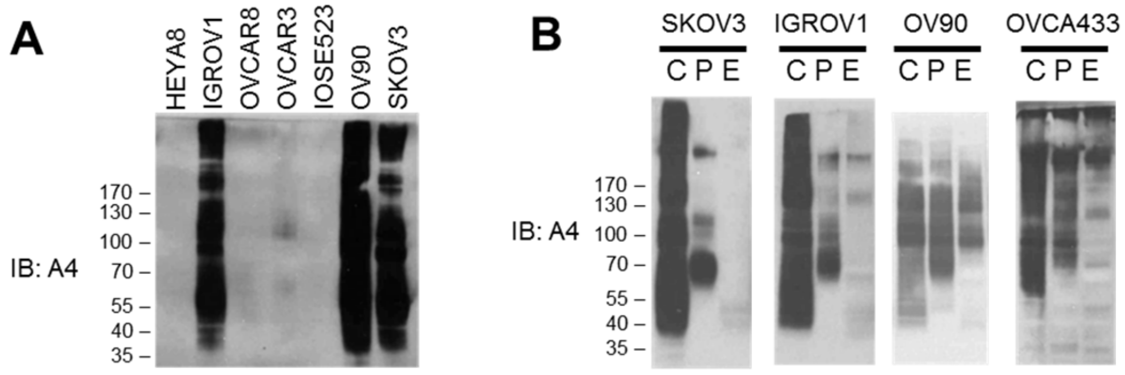

Supplementary Figure 5

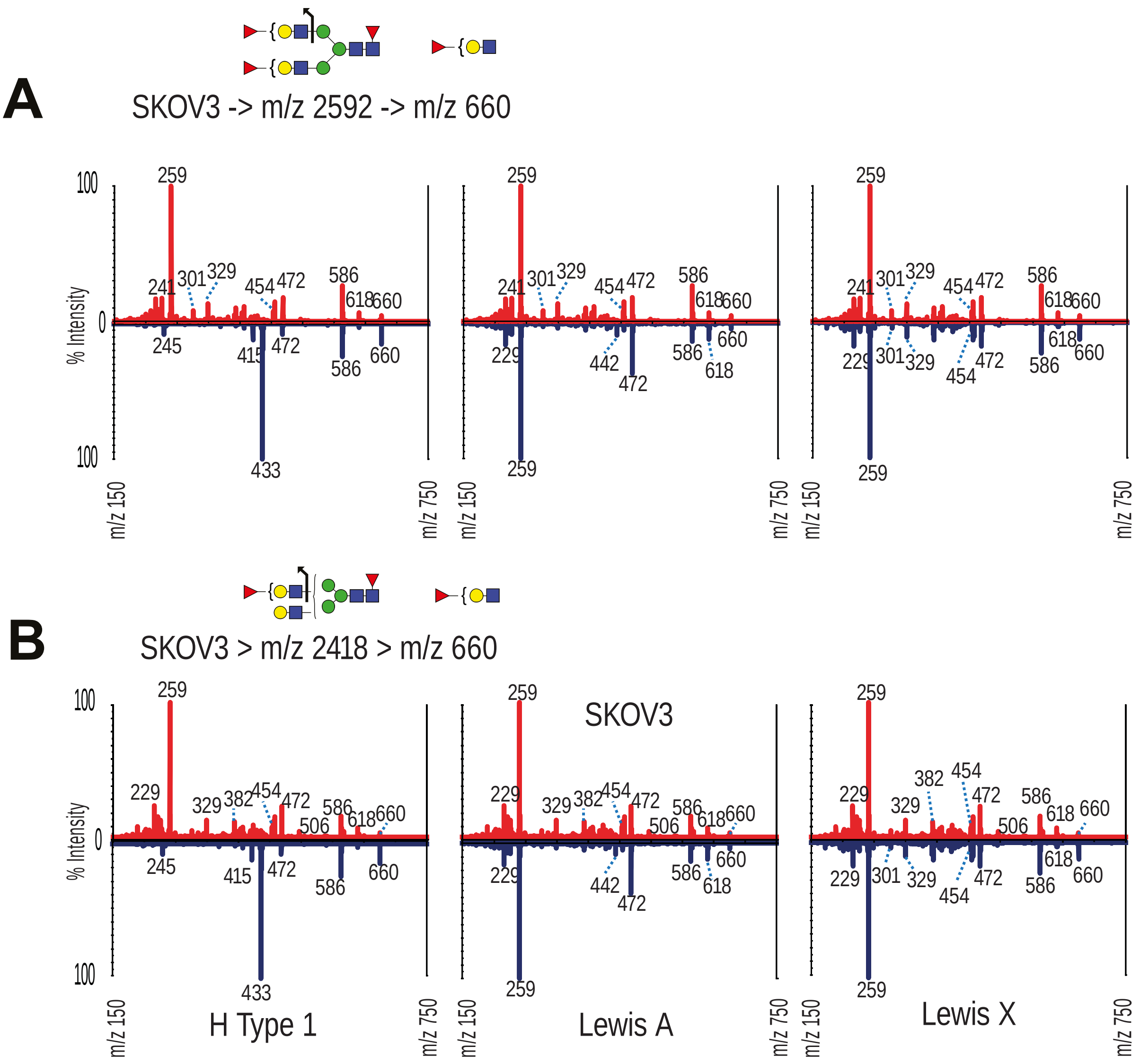

C

Fragment (m/z)	H1 standard (LNFP1)	LeA standard (LNFP2)	LeX standard (mouse kidney)	m/z 2592 (SKOV3)	m/z 2418 (SKOV3)
454/472	0.000	0.054	0.277	0.416	0.303
433	✓	x	x	x	x
329	x	x	✓	✓	✓

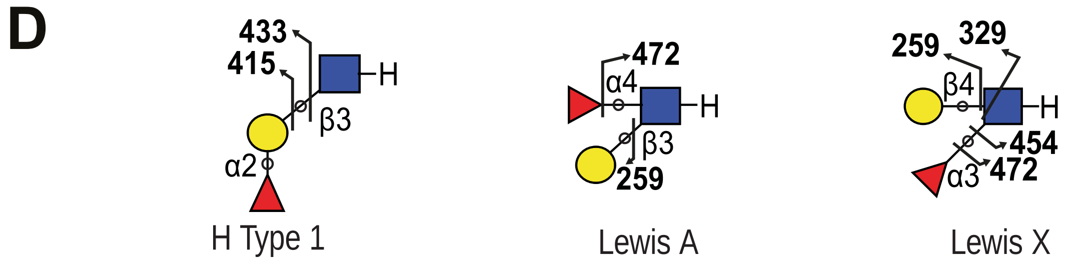

Supplementary Figure 6

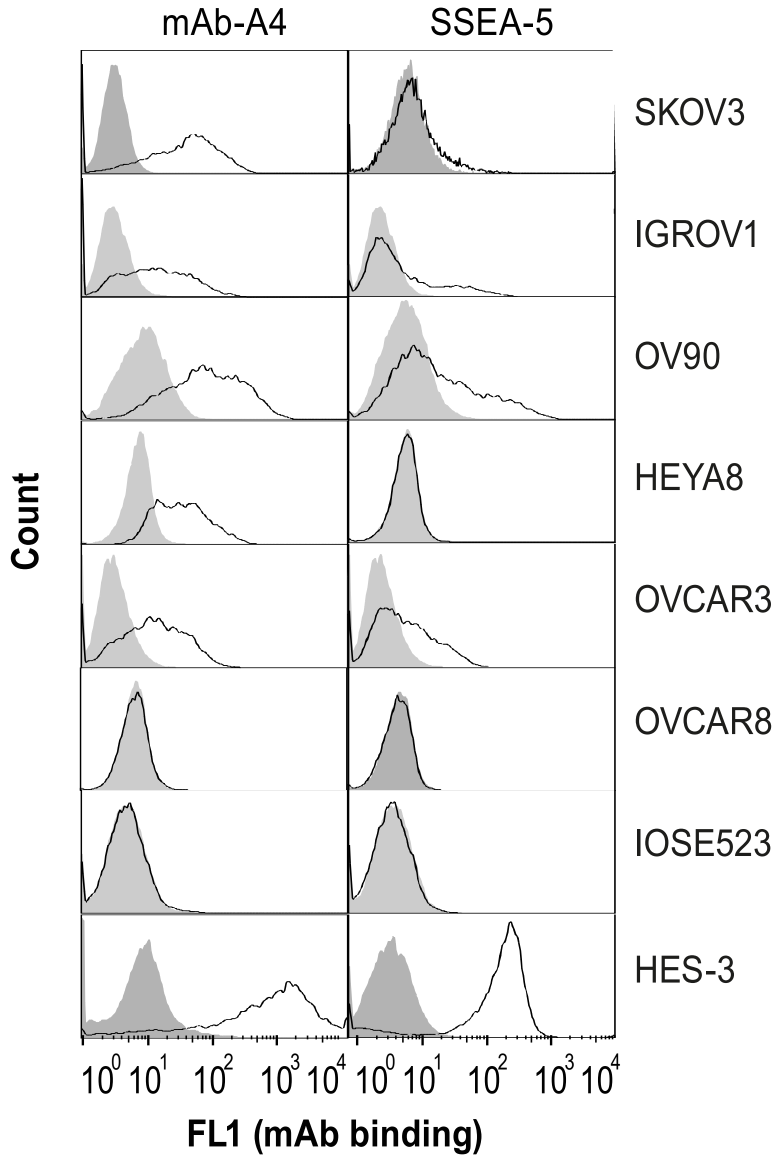

Supplementary Figure 7

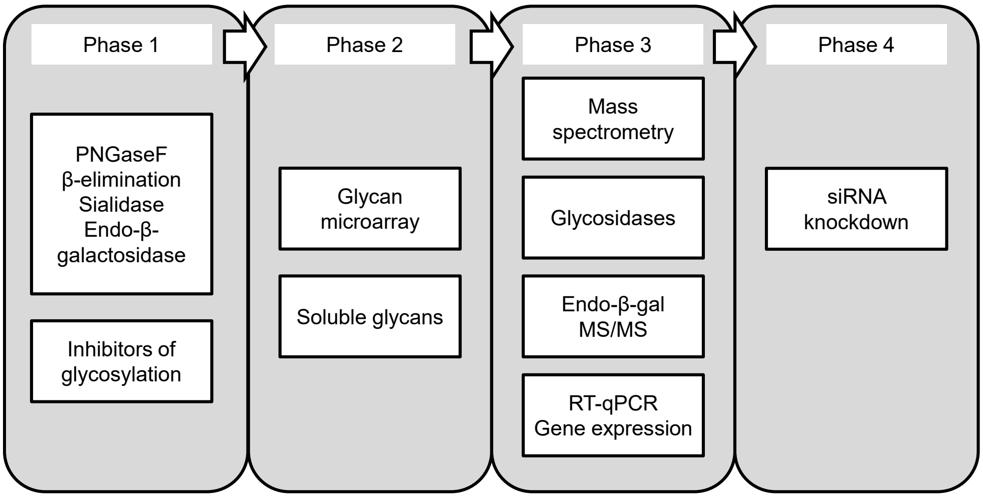

REFERENCES

1. Tan, T. Z., Miow, Q. H., Huang, R. Y.-J., Wong, M. K., Ye, J., Lau, J. A., Wu, M. C., Bin Abdul Hadi, L. H., Soong, R., Choolani, M., Davidson, B., Nesland, J. M., Wang, L.-Z., Matsumura, N., Mandai, M., Konishi, I., Goh, B.-C., Chang, J. T., Thiery, J. P., and Mori, S. (2013) Functional genomics identifies five distinct molecular subtypes with clinical relevance and pathways for growth control in epithelial ovarian cancer. *EMBO Mol. Med.* **5**, 983–998
2. Domon, B., and Costello, C. E. (1988) A systematic nomenclature for carbohydrate fragmentations in FAB-MS/MS spectra of glycoconjugates. *Glycoconj. J.* **5**, 397–409
3. Spina, E., Sturiale, L., Romeo, D., Impallomeni, G., Garozzo, D., Waidelich, D., and Glueckmann, M. (2004) New fragmentation mechanisms in matrix-assisted laser desorption/ionization time-of-flight/time-of-flight tandem mass spectrometry of carbohydrates. *Rapid Commun. Mass Spectrom. RCM.* **18**, 392–398
4. García-Vallejo, J. J., Van Dijk, W., Van Het Hof, B., Van Die, I., Engelse, M. A., Van Hinsbergh, V. W. M., and Gringhuis, S. I. (2006) Activation of human endothelial cells by tumor necrosis factor-α results in profound changes in the expression of glycosylation-related genes. *J. Cell. Physiol.* **206**, 203–210
5. Fukuda, M. N., and Matsumura, G. (1976) Endo-beta-galactosidase of Escherichia freundii. Purification and endoglycosidic action on keratan sulfates, oligosaccharides, and blood group active glycoprotein. *J. Biol. Chem.* **251**, 6218–6225
6. Scudder, P., Hanfland, P., Uemura, K., and Feizi, T. (1984) Endo-beta-D-galactosidases of Bacteroides fragilis and Escherichia freundii hydrolyze linear but not branched oligosaccharide domains of glycolipids of the neolacto series. *J. Biol. Chem.* **259**, 6586–6592
